# Riluzole for Degenerative Cervical Myelopathy: A Secondary Analysis of the CSM-PROTECT Trial

**DOI:** 10.1001/jamanetworkopen.2024.15643

**Published:** 2024-06-21

**Authors:** Michael G. Fehlings, Karlo M. Pedro, Mohammed Ali Alvi, Jetan H. Badhiwala, Henry Ahn, H. Francis Farhadi, Christopher I. Shaffrey, Ahmad Nassr, Praveen Mummaneni, Paul M. Arnold, W. Bradley Jacobs, K. Daniel Riew, Michael Kelly, Darrel S. Brodke, Alexander R. Vaccaro, Alan S. Hilibrand, Jason Wilson, James S. Harrop, S. Tim Yoon, Kee D. Kim, Daryl R. Fourney, Carlo Santaguida, Eric M. Massicotte, Peng Huang

**Affiliations:** 1 Division of Neurosurgery, Toronto Western Hospital, University of Toronto, Toronto, Ontario, Canada; 2 Division of Neurosurgery and Spine Program, Department of Surgery, University of Toronto, Toronto, Ontario, Canada; 3 Institute of Medical Science, University of Toronto, Toronto, Ontario, Canada; 4 Division of Orthopaedic Surgery, St Michael’s Hospital, University of Toronto, Toronto, Ontario, Canada; 5 Department of Neurological Surgery, Ohio State University, Columbus; 6 Department of Neurosurgery, University of Virginia, Charlottesville; 7 Department of Orthopedic Surgery, Mayo Clinic, Rochester, Minnesota; 8 Department of Neurosurgery, University of California, San Francisco; 9 Department of Neurosurgery, Kansas University Medical Center, Kansas City; 10 Department of Clinical Neurosciences, University of Calgary, Calgary, Alberta, Canada; 11 Department of Orthopedic Surgery, Columbia University, New York, New York; 12 Department of Orthopaedic Surgery, University of California, San Diego; 13 Department of Orthopaedics, University of Utah, Salt Lake City; 14 Department of Orthopaedic Surgery, Rothman Orthopaedic Institute, Thomas Jefferson University, Philadelphia, Pennsylvania; 15 Department of Neurosurgery, Louisiana State University, New Orleans; 16 Department of Neurological Surgery, Thomas Jefferson University, Philadelphia, Pennsylvania; 17 Department of Orthopaedics, Emory University, Atlanta, Georgia; 18 Department of Neurological Surgery, University of California, Davis, Sacramento; 19 Division of Neurosurgery, University of Saskatchewan, Saskatoon, Saskatchewan, Canada; 20 Department of Neurology and Neurosurgery, McGill University Health Centre, Montreal, Quebec, Canada; 21 Department of Oncology, Johns Hopkins University, Baltimore, Maryland; 22 Department of Biostatistics, Johns Hopkins University, Baltimore, Maryland

## Abstract

**Importance:**

The modified Japanese Orthopaedic Association (mJOA) scale is the most common scale used to represent outcomes of degenerative cervical myelopathy (DCM); however, it lacks consideration for neck pain scores and neglects the multidimensional aspect of recovery after surgery.

**Objective:**

To use a global statistical approach that incorporates assessments of multiple outcomes to reassess the efficacy of riluzole in patients undergoing spinal surgery for DCM.

**Design, Setting, and Participants:**

This was a secondary analysis of prespecified secondary end points within the Efficacy of Riluzole in Surgical Treatment for Cervical Spondylotic Myelopathy (CSM-PROTECT) trial, a multicenter, double-blind, phase 3 randomized clinical trial conducted from January 2012 to May 2017. Adult surgical patients with DCM with moderate to severe myelopathy (mJOA scale score of 8-14) were randomized to receive either riluzole or placebo. The present study was conducted from July to December 2023.

**Intervention:**

Riluzole (50 mg twice daily) or placebo for a total of 6 weeks, including 2 weeks prior to surgery and 4 weeks following surgery.

**Main Outcomes and Measures:**

The primary outcome measure was a difference in clinical improvement from baseline to 1-year follow-up, assessed using a global statistical test (GST). The 36-Item Short Form Health Survey Physical Component Score (SF-36 PCS), arm and neck pain numeric rating scale (NRS) scores, American Spinal Injury Association (ASIA) motor score, and Nurick grade were combined into a single summary statistic known as the global treatment effect (GTE).

**Results:**

Overall, 290 patients (riluzole group, 141; placebo group, 149; mean [SD] age, 59 [10.1] years; 161 [56%] male) were included. Riluzole showed a significantly higher probability of global improvement compared with placebo at 1-year follow-up (GTE, 0.08; 95% CI, 0.00-0.16; *P* = .02). A similar favorable global response was seen at 35 days and 6 months (GTE for both, 0.07; 95% CI, −0.01 to 0.15; *P* = .04), although the results were not statistically significant. Riluzole-treated patients had at least a 54% likelihood of achieving better outcomes at 1 year compared with the placebo group. The ASIA motor score and neck and arm pain NRS combination at 1 year provided the best-fit parsimonious model for detecting a benefit of riluzole (GTE, 0.11; 95% CI, 0.02-0.16; *P* = .007).

**Conclusions and Relevance:**

In this secondary analysis of the CSM-PROTECT trial using a global outcome technique, riluzole was associated with improved clinical outcomes in patients with DCM. The GST offered probability-based results capable of representing diverse outcome scales and should be considered in future studies assessing spine surgery outcomes.

## Key Points

**Question:**

Can a composite outcome be used to represent the diverse aspects of functional recovery in patients with degenerative cervical myelopathy (DCM) to assess the potential efficacy of perioperative riluzole in optimizing recovery?

**Findings:**

In this secondary analysis of a randomized clinical trial including 290 surgical patients with DCM, use of a global statistical approach combining 5 distinct outcome scales revealed a superior global treatment effect with riluzole compared with placebo, with a statistically significant difference in clinical improvement at 1-year follow-up.

**Meaning:**

In this study, treatment with riluzole was associated with an improved composite outcome among patients with DCM managed surgically.

## Teaser

This secondary analysis of a randomized clinical trial uses a global statistical test to assess composite clinical outcomes of perioperative riluzole vs placebo for patient recovery after spinal surgery for degenerative cervical myelopathy.

## Introduction

Degenerative cervical myelopathy (DCM) is an age-related, progressive spinal disorder affecting approximately 600 per 1 million individuals in North America.^1^ The burden of DCM-related hospitalizations, diminished productivity, and disability payments collectively contribute to an annual health care cost exceeding £681 million in the UK.^2^ Despite DCM being the most prevalent cause of nontraumatic spinal cord impairment among adults,^1^ treatment options to enhance neurologic recovery are lacking. Consequently, developing therapeutic strategies to improve DCM outcomes is an urgent research priority.

Informed by preclinical data suggesting that the sodium-glutamate antagonist riluzole, an approved drug that attenuates neurodegeneration in amyotrophic lateral sclerosis, improves neurologic outcomes in preclinical DCM models,^3,4,5^ the Efficacy of Riluzole in Surgical Treatment for Cervical Spondylotic Myelopathy (CSM-PROTECT) trial was conducted to evaluate the effect of riluzole on outcomes among patients undergoing surgical treatment for DCM. In 2021, the trial results were published and indicated that riluzole did not yield improvement in scores on the modified Japanese Orthopaedic Association (mJOA) scale, which was the study’s primary end point.^6^ However, limitations of the mJOA scale in assessing key outcomes in DCM, including neck pain, have been raised.^7,8,9^ Some patients in the trial also reached the ceiling threshold of the mJOA scale, potentially masking significant improvements achieved with riluzole. Additionally, a substantial number of patients showed improvement on multiple prespecified secondary trial end points, including reductions in neck and arm pain scores at 1 year of follow-up. Consequently, further investigation is warranted to identify a suitable scale capable of capturing clinically significant treatment effects across various end points.

In DCM studies, single primary outcome measures, such as the mJOA scale or 36-Item Short Form Health Survey (SF-36) questionnaire, are traditionally favored for evaluating interventions. However, this approach may not capture the multifaceted impact of therapies adequately. For instance, using the mJOA scale as the solitary primary outcome measure disregards the inherent heterogeneity in DCM phenotypes and neglects other aspects of recovery, such as improvements in quality of life, disability, and pain. In such contexts, an assessment that combines multiple relevant outcomes could offer a more comprehensive perspective on treatment efficacy.

A novel statistical approach gaining traction in studies with multiple end points is the application of a global statistical test (GST), initially proposed by O’Brien^10^ and subsequently refined by Huang et al.^11^ This innovative technique uses a nonparametric method based on a composite rank sum of diverse variables, offering a single test for a global interpretation on recommending a new treatment. Clinical interest in this technique has increased, particularly in the study of complex neurologic disorders, such as stroke^12^ and Parkinson disease,^13^ for which treatment influence spans multiple facets of recovery.

Multiple prior secondary analyses have consistently demonstrated the superior sensitivity to treatment effects of GST compared with alternative methods.^14,15,16^ Despite its compelling features, the application of GST in spinal research is underexplored. To our knowledge, no quantitative research to date has interrogated the diagnostic utility of GST in patients with DCM. In this study, we applied the GST technique to reanalyze data from the CSM-PROTECT trial. Given the favorable effects of riluzole on various secondary outcome measures in the trial, we hypothesized that the adjuvant use of riluzole would yield superior global outcomes compared with placebo in patients with DCM undergoing decompressive surgery.

## Methods

### Data Source and Patient Population

The CSM-PROTECT trial (NCT01257828) was a multicenter, double-blind, phase 3 randomized clinical trial conducted between January 2012 and May 2017. The CSM-PROTECT protocol^5^ and CSM-PROTECT trial results^6^ have been reported previously. From July to December 2023, we performed a post hoc secondary analysis of predefined secondary end points in all patients with a final diagnosis of DCM. Inclusion criteria specified patient age between 18 and 80 years and an mJOA scale score between 8 and 14 points, indicative of moderate to severe functional disability. All patients underwent decompressive surgery followed by postsurgical rehabilitation in accordance with local institutional protocols. The trial protocol (Supplement 1) was approved by the research board at each participating center. Patient informed consent was waived because this was a reanalysis of a previously collected dataset; however, stringent confidentiality was ensured throughout the analysis. This study followed the Consolidated Standards of Reporting Trials (CONSORT) reporting guideline.

### Study Intervention and Clinical Assessment

All patients in the treatment arm of the CSM-PROTECT trial received oral riluzole, 50 mg twice daily, for 14 days before surgery and for an additional 28 days after surgery for a total of 6 weeks. Clinical evaluation used the mJOA scale score as the primary outcome of the study. Secondary a priori end points included the Nurick grade and Neck Disability Index to measure functional status and disability, American Spinal Injury Association (ASIA) motor and sensory score to measure neurologic function, European Quality of Life 5-Dimension questionnaire (EQ-5D) and SF-36 Physical Component Summary (SF-36 PCS) scores to measure quality of life, and both the arm and the neck pain numeric rating scale (NRS) scores to measure pain outcomes.

### Sample Selection and Missing Data Management

Patients in both the riluzole and the placebo groups were considered in this secondary analysis. We retained the original randomization in accordance with the intention-to-treat principle. To address missing follow-up data, a multiple imputation procedure with 10 iterations was used, similar to the original trial.^6^ No systematic patterns for missing values were found with respect to the study variables; thus, the missing-at-random assumption was deemed to be plausible.

### Outcome Measure and Ancillary Analysis

In this post hoc secondary analysis, our primary focus was to assess clinical improvement at 1-year follow-up after surgery using 5 secondary outcome scales from the CSM-PROTECT trial. We used a global statistical test (GST) to evaluate the overall impact of riluzole treatment across SF-36 PCS, neck pain NRS, arm pain NRS, ASIA motor score, and Nurick grade, yielding a summary statistic known as the global treatment effect (GTE).^17^ These selected measurement scales offer distinct insights into quality of life, somatic pain, neurologic deficit, and functional status, collectively addressing the multiple dimensions of recovery among patients with DCM.

The calculated GTE was further analyzed by tracking changes at 35 days, 6 months, and 1 year. Additionally, an exploratory analysis incorporated different combinations of outcome scales into the GST model to elucidate salient properties of the GST approach.

### Statistical Analysis

All analyses were conducted using the GST function as described by Huang et al.^18^ The GST involves 2 key components: GTE calculation and hypothesis testing. To perform the GST, data were consistently coded to ensure that larger values indicated better outcomes for all measures. The data were structured into a matrix with columns representing the 5 distinct outcome scales and rows containing the ranked observations (final minus baseline raw scores) for each patient. The total ranks from the placebo and riluzole groups were then compared using a 2-sample *t* test with the appropriate adjustment factor for sample variance.^11^ The resulting *t* statistics were compared with the critical value in the *t* value distribution table.

To calculate the GTE, θ values (θ*_ν_*) for each outcome measure were initially derived using the following formula^18^:
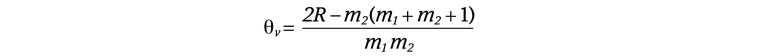
where *m*_1_ is the number of patients in the placebo group, *m*_2_ is the number of patients in the riluzole group, and *R* is the sum of the total ranks from all patients in the riluzole group. The θ values represent a measure of net gain (*A* − *B*), where *A* is the probability that treatment is better than placebo and *B* is the probability that placebo is better than treatment. All calculated θ values were then aggregated, and their average (eg, Σθ*_ν_/*5) yielded a single metric, the GTE.^17^ The interpretation of GTE values adhered to a previously established guideline^18^: GTE of 0 (ie, *A* = *B*) indicates no global difference between the riluzole and placebo groups, GTE greater than 0 (ie, *A* > *B*) signifies that the riluzole group achieved globally superior outcome values compared with the placebo group, and GTE less than 0 (ie, *A* < *B*) suggests that the riluzole group achieved globally inferior outcome values compared with the placebo group. The final GTE value could then be transformed into a probability of improved global outcomes using the formula (1 + GTE)/2.^17^ A conservative estimate (upper bound) of the GTE SD was computed using the following formula:
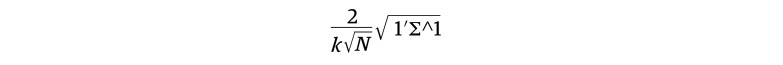
where *k* is the number of end points, *N* is the total sample size, and the matrix Σ^ is as defined by Huang et al.^17^ The GTE estimation and hypothesis testing were conducted using R, version 4.2.1 (R Project for Statistical Computing). All tests were 1-sided, and statistical significance was defined as *P* < .025, aligning with the original trial protocol and the prescribed use of the GST analysis.

## Results

### Baseline Characteristics

**Figure 1.**
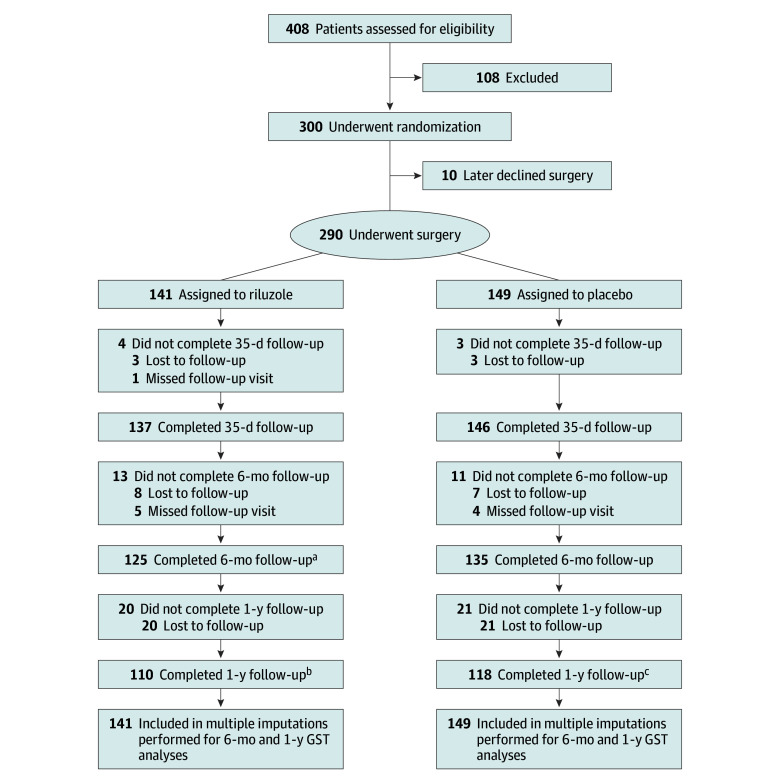
Flowchart Depicting the Patient Recruitment and Randomization Strategy in the CSM-PROTECT Trial CSM-PROTECT indicates Efficacy of Riluzole in Surgical Treatment of Cervical Spondylotic Myelopathy; GST, global statistical test. ^a^Includes 1 patient who missed the 35-day follow-up visit. ^b^Includes 5 patients who missed the 6-month follow-up visit. ^c^Includes 4 patients who missed the 6-month follow-up visit.

A total of 290 patients with a mean (SD) age of 59 (10.1) years (129 [44%] female; 161 [56%] male) underwent surgery and were randomized into the riluzole or placebo group. Of these, 149 patients received the placebo, while 141 were allocated to riluzole treatment (Figure 1). The demographic and clinical profile of these 290 patients in the intention-to-treat analysis has been extensively detailed previously.^6^ Baseline variables including surgical procedure and fusion levels were well balanced between the 2 treatment arms. Gait impairment was the most common preoperative symptom, seen in 146 patients (50%). The mean (SD) duration from symptom onset to study enrollment was 38 (60) months. Imaging studies consistently identified spondylosis and disc prolapse as the predominant pathologic entities. The overall follow-up rate was 79% (228 of 290) at 1 year and, with imputation techniques, was enhanced to 100%.

### Clinical Outcomes

**Figure 2.**
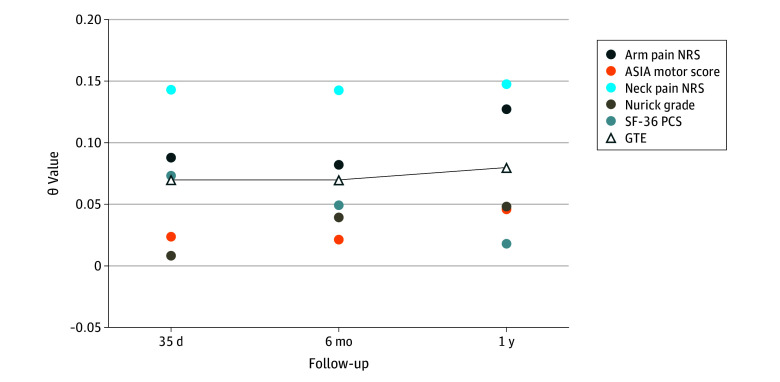
Application of the Global Statistical Test in the Estimation of Global Treatment Effect (GTE) Each θ value (θ*_v_*) quantifies the difference between the probability that treatment is better than control (*A*) and the probability that control is better than treatment (*B*) (ie, θ*_v_* = [*A* − *B*]). ASIA indicates American Spinal Injury Association; NRS, numeric rating scale; SF-36 PCS, 36-Item Short Form Health Survey Physical Component Summary.

The evaluation of outcomes of riluzole using the global statistical approach is summarized in Figure 2 and the eTable in Supplement 2. At the 1-year primary outcome assessment, the placebo group had a median rank sum of 701 (IQR, 522-848), while the riluzole group showed a higher median rank sum of 744 (IQR, 584-958), with a higher summed rank signifying improved outcomes. Using a modified O’Brien nonparametric test,^10^ a *t* statistic of 2.06 was obtained, exceeding the critical value of 1.97 (1-sided significance, α = .025; *df* = 288). In terms of GTE calculation, all θ values for the 5 outcome measures at 1 year were positive, with neck pain reaching a maximum θ value of 0.15. The final GTE score of 0.08 (95% CI, 0.00-0.16; *P* = .02) suggests that riluzole-treated patients had a 54% probability of achieving favorable outcomes compared with the placebo group.

A parallel trend emerged at 35 days and at 6 months, with the riluzole group exhibiting a greater median rank sum compared with the placebo group (35 days: 740 [IQR, 606-910] for riluzole vs 696 [IQR, 514-888] for placebo; 6 months: 762 [IQR, 576-946] for riluzole vs 714 [IQR, 510-889] for placebo; both *P* = .04). The GTE at both 35 days and 6 months (0.07; 95% CI, −0.01 to 0.15; *P* = .04), while positive, did not indicate a statistically significant benefit of riluzole treatment at these early time points.

**Figure 3.**
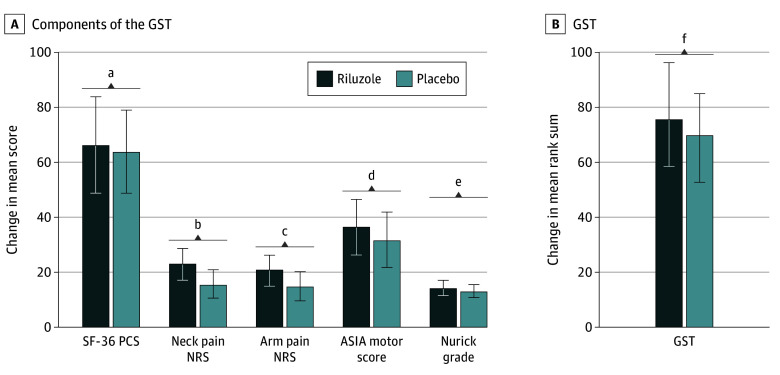
Components of the Global Statistical Test (GST) Used in the Secondary Analysis of the CSM-PROTECT Trial The mean change from baseline to 1 year is presented for each of the 5 components (A) and the GST (B). Error bars indicate 95% CIs. CSM-PROTECT indicates Efficacy of Riluzole in Surgical Treatment of Cervical Spondylotic Myelopathy. ^a^*P* = .80 by Wilcoxon test; *P* = .42 by *t* test. ^b^*P* = .02 by Wilcoxon test; *P* = .01 by *t* test. ^c^*P* = .05 by Wilcoxon test; *P* = .04 by *t* test. ^d^*P* = .42 by Wilcoxon test; *P* = .23 by *t* test. ^e^*P* = .45 by Wilcoxon test; *P* = .20 by *t* test. ^f^*P* = .02, calculated using a 2-sample *t* test comparing the mean rank sum between riluzole and placebo groups.

Normality tests using Q-Q plots (eFigure in Supplement 2) showed that with the exception of SF-36 PCS, all outcomes deviated from a normal distribution, justifying the use of nonparametric approaches in the analysis. Univariate tests used the Wilcoxon test to compare the riluzole and placebo groups across the 5 outcomes (Figure 3), with additional *P* values from *t* tests provided for comparison. While this analysis revealed improvements in all 5 outcomes with riluzole, none reached statistical significance in the univariate test following Bonferroni adjustment for familywise error rate (ie, *P* < .01). In contrast, the GST showed a significant difference, with a higher rank sum in the riluzole group compared with the placebo group.

### GST Subset Analyses

Table 1 and Table 2 present the GTE at 1 year under different combinations of outcome scales, highlighting some key properties of the GST approach. As previously noted, the original 5-outcome combination yielded a GTE of 0.08 (95% CI, 0.00-0.16; *P* = .02). Replacing the ASIA motor score with the ASIA sensory score, characterized by a θ value of 0.02, reduced the GTE to 0.07 (95% CI, −0.01 to 0.15; *P* = .03). A similar reduction in GTE to 0.04 (95% CI, −0.04 to 0.12; *P* = .27) was observed when the mJOA scale score replaced the Nurick score in the 5-outcome scale. Replacing neck pain NRS with the NDI score and arm pain NRS with ASIA sensory score further decreased the GTE to 0.02 (95% CI, −0.06 to 0.10; *P* = .28), underscoring the value of these scores in estimating the therapeutic effect of riluzole.

**Table 1.** Variations in GTE Values Across Different Outcome Scale Combinations at 1 Year for 5-Outcome Combinations

**Combination**	**Outcome scale,** θ*_v_*					**GTE (95% CI)**	***P* value**
1							
Scales	SF-36 PCS	Neck pain NRS	Arm pain NRS	ASIA motor score	Nurick grade	0.08 (0.00 to 0.16)	.02
θ*_v_*	0.02	0.15	0.13	0.05	0.05		
2							
Scales	SF-36 PCS	Neck pain NRS	Arm pain NRS	ASIA sensory score	Nurick grade	0.07 (−0.01 to 0.15)	.03
θ*_v_*	0.02	0.15	0.13	0.02	0.05		
3							
Scales	SF-36 PCS	Neck pain NRS	Arm pain NRS	ASIA motor score	mJOA scale	0.04 (−0.04 to 0.12)	.27
θ*_v_*	0.02	0.15	0.13	0.05	−0.13		
4							
Scales	SF-36 PCS	NDI score	ASIA sensory score	ASIA motor score	Nurick grade	0.02 (−0.06 to 0.10)	.28
θ*_v_*	0.02	−0.02	0.02	0.05	0.05		

Abbreviations: ASIA, American Spinal Injury Association; GTE, global treatment effect; mJOA, modified Japanese Orthopaedic Association; NDI, Neck Disability Index; NRS, numeric rating scale; SF-36 PCS, 36-Item Short Form Health Survey Physical Component Summary.

**Table 2.** Variations in GTE Values Across Different Outcome Scale Combinations at 1 Year for 3-Outcome Combinations

**Combination**	**Outcome scale,** θ*_v_*			**GTE (95% CI)**	***P* value**
1					
Scales	ASIA motor score	Neck pain NRS	Arm pain NRS	0.11 (0.02 to 0.16)	.007
θ*_v_*	0.05	0.15	0.13		
2					
Scales	ASIA motor score	Neck pain NRS	SF-36 PCS	0.07 (−0.02 to 0.16)	.05
θ*_v_*	0.05	0.15	0.02		
3					
Scales	ASIA motor score	Neck pain NRS	EQ-5D	0.06 (−0.03 to 0.15)	.10
θ*_v_*	0.05	0.15	−0.02		

Abbreviations: ASIA, American Spinal Injury Association; EQ-5D, European Quality of Life 5-Dimension questionnaire; GTE, global treatment effect; NRS, numeric rating scale; SF-36 PCS, 36-Item Short Form Health Survey Physical Component Summary.

The highest GTE value (mean, 0.11; 95% CI, 0.02-0.16; *P* = .007) was achieved by using the triple combination of the ASIA motor score, neck pain NRS, and arm pain NRS (Table 2). This parsimonious model provided the highest power for detecting differences between the riluzole and placebo groups. The remaining 2 iterations of the 3-outcome combination revealed the importance of the common dose-effect assumption (eg, consistent effect on all outcomes), a key element in GST calculation. To assess the impact of violating this assumption, we replaced the SF-36 PCS score (θ, 0.02) with EQ-5D scores at 1 year (θ, −0.02), resulting in a decrease in GTE from 0.07 (95% CI, −0.02 to 0.16; *P* = .05) to 0.06 (95% CI, −0.03 to 0.15; *P* = .10).

## Discussion

This secondary analysis of the CSM-PROTECT trial that used a global statistical approach found a favorable treatment effect with riluzole compared with placebo at 1 year for patients undergoing surgery for DCM. Our study not only demonstrated the utility of this novel statistical approach for assessing multiple trial end points after spine surgery but also, given the salient findings, offered justification for considering riluzole as an adjunctive treatment option for DCM, a condition currently devoid of therapeutic pharmacologic neuroprotective or neuroregenerative approaches.

This study supported the robustness and computational feasibility of the GST in evaluating the impact of riluzole across diverse outcome measures. Positive θ values for the SF-36 PCS, ASIA motor score, and Nurick grade in addition to neck and arm pain scores at 35 days, 6 months, and 1 year indicated consistent beneficial treatment effects, resulting in a positive GTE value. Despite nonsignificant *P* values in the univariate *t* test with Bonferroni adjustment for each of the 5 outcome scales, the GST yielded significant results at 1-year follow-up, suggesting its superior statistical power in assessing correlated outcomes. The observed positive GTE at 35 days and 6 months (GTE of 0.07 for both), although not statistically significant, aligned with 1-year findings indicating a moderate positive effect of riluzole. Interestingly, the neck pain score consistently had the highest θ value at all time points, showing a substantial association of riluzole with this measure, supported by the initial CSM-PROTECT trial results^6^ and basic science studies.^4,19^ In particular, the ability of riluzole to attenuate glutaminergic excitotoxicity has been proposed to be the main mechanism for alleviating the neuropathic component of neck pain in patients with DCM.^4,19^ Our secondary analysis suggests that future riluzole studies may benefit from using the ASIA motor score and neck and arm pain NRS as primary trial end points given that their collective assessment yielded the highest GTE value and highest power to detect outcome differences in our subset analysis.

Our findings highlight the inadequacy of using a single outcome measure to fully capture the association of riluzole with the multiple aspects of recovery in patients with DCM. It is now recognized that DCM progression varies across physical functioning, neurologic deficits, and disability domains, resulting in diverse patient phenotypes and recovery trajectories.^7,20^ The limitations of categorizing outcomes into mild, moderate, and severe using the mJOA scale alone are evident, particularly because it does not encompass neck pain, an identified factor associated with quality of life and a top recovery priority in patients with DCM.^9^ To address these issues, the integration of multiple measures may have utility as a strategy.

The choice of the GST in this secondary analysis was based on several key advantages.^21,22,23^ The robustness of the GST with small sample sizes, applicability in the absence of normal distribution assumptions, and ability to integrate diverse end points on different scales made it ideal for our study.^10,24^ This is particularly pertinent as the primary outcome included both continuous variables (ie, SF-36 PCS) and ordinal categorical variables (ie, NRS and Nurick grade). Notably, the GTE facilitates estimation of the probability of clinical improvement, offering practical utility in clinical counseling.

In contrast to the Hotelling *T*^2^ and Bonferroni tests, which detect differences between 2 groups regardless of treatment effect, the GST approach was designed to test a directional hypothesis about which treatment is superior.^24^ For instance, if a treatment is significantly better in 1 end point but equally significantly worse in a second end point, the Hotelling *T*^2^ and Bonferroni tests may yield small *P* values, indicating that treatment is statistically significantly different but failing to indicate which treatment is better. Conversely, in such cases, the GTE would be 0 with a nonsignificant GST *P* value, providing an assessment of no GTE and thereby offering a more rational treatment recommendation. The GST approach may contribute to a unified global approach in DCM research.^25,26^

Our study emphasizes the importance of alternative analytical methods in clinical trial evaluation, revealing effects not evident with conventional models.^27^ The traditional solitary end point approach in DCM research may potentially overlook clinically significant treatment effects, especially when a patient’s response to treatment can manifest in many distinct forms. Our study found that application of a global outcome technique in the analysis of spine trial data may provide a rationale for clinicians to consider the option of using riluzole as an adjunctive treatment for DCM, particularly to enhance clinical outcomes during the perioperative and postoperative recovery periods. Our findings of a significant association of riluzole with the studied end points may impact its clinical use, potentially positioning it as a neuroprotective option for DCM.^28,29,30^ However, further research is crucial to validate and replicate these results.

### Limitations

Several potential limitations of this study should be considered. The selection of the 5 outcome measurement scales for GST computation may have introduced intrinsic bias. To address this, we prespecified these outcomes before statistical analysis and performed various sensitivity analyses, consistently yielding positive GTE values. We acknowledge that this study represents a reanalysis of the original CSM-PROTECT trial data. The goal was to complement the initial findings of a significant effect of riluzole on neck pain and to assess other aspects of recovery. In conducting this analysis, we applied a rigorous statistical approach, adhering to the same statistical level of significance, intention-to-treat analysis, and imputation method used in the CSM-PROTECT trial, ensuring methodologic consistency.

## Conclusions

In this secondary analysis of the CSM-PROTECT trial using a global outcome technique, riluzole was associated with improved clinical outcomes in patients with DCM at 1-year follow-up. This observation was based on the collective improvement across multiple end points, as opposed to the conventional reliance on a single outcome measure. The findings suggest that use of the GST model may substantially improve understanding of the multiple domains of recovery in patients with DCM.

## Notes

Supplement 1.
Trial Protocol

Supplement 2. **eFigure.** Q-Q Plots of 1-Year Change From Baseline to Examine Data Normality **eTable.** Application of the GST in the Estimation of GTE

Supplement 3.
Data Sharing Statement
