# Supplementary material for: Riluzole for Degenerative Cervical Myelopathy: A Secondary Analysis of the CSM-PROTECT Trial
Source: JAMA Netw Open. 2024 Jun 21;7(6):e2415643. doi: 10.1001/jamanetworkopen.2024.15643 (PMC11193126; doi:10.1001/jamanetworkopen.2024.15643)
Supplement: Supplement 1. — Trial Protocol [file jamanetwopen-e2415643-s001.pdf]

Efficacy of Riluzole in Patients with Cervical Spondylotic Myelopathy Undergoing Surgical Treatment

A Randomized, Double-Blind, Placebo-Controlled, Multi-Center Study

Sponsor
AOSpine North America

Version 2.0: February 25, 2013

Reference Number: SPN-10-001

The information provided in this document is strictly confidential and is available for review to investigators, potential investigators, investigational review boards and regulatory authorities.

1 Investigator Statement 6
2 Synopsis 7
3 Background Information 11
3.1 Justification for Investigation 11
3.2 Riluzole 11
3.2.1 Pharmacology of Riluzole 12
3.2.2 Regulatory Status 12
3.3 Study Scope and Duration 13
4 Design of the Study 15
4.1 Objectives of the Study 15
4.1.1 Primary Endpoint 15
4.1.2 Secondary Efficacy Endpoints 15
4.1.3 Other Endpoints 15
4.1.4 Safety Evaluation 16
4.2 Selection of Investigators 16
4.3 Inclusion Criteria 16
4.4 Exclusion Criteria 17
4.5 Study Procedures 18
4.5.1 Study Schematic 18
4.5.2 Study Time Line 21
4.5.3 Enrollment and Randomization 26
4.5.4 Study Drug Procedures 26
4.5.5 Surgical Procedure 27
4.5.6 Post-surgical Rehabilitation and Procedures 27
4.5.7 Clinical Laboratory Procedures 28
4.6 Subjects Completion and Disposition 28
4.6.1 Screen Failure 28
4.6.2 Enrolled Subject 28
4.6.3 Withdrawn Subject 28
4.6.4 Lost to Follow-up 29
4.6.5 Completed Subject 29

5 Statistical Analysis 30
5.1 Primary Efficacy Endpoint 30
5.2 Secondary Efficacy Endpoints 30
5.3 Other Endpoints 30
5.3.1 Complications and AE 31
5.4 Study success 31
5.4.1 Main Definition of Success 31
5.4.2 Alternative Definition of Success 31
5.5 Statistical Testing of Primary Endpoint (mJOA) 31
5.6 Statistical Testing of Secondary Endpoints 32
5.7 Sample Size Estimate 32
5.8 Statistical Performance and Simulations 33
5.9 Adaptive Design and Sample Size Re-estimate 37
5.10 Analysis Populations 37
5.10.1 Intention-to-Treat 37
5.10.2 Modified Intention-to-Treat 37
5.10.3 Per-protocol Population 37
5.10.4 Completed Subjects population 37
5.10.5 Primary analysis population 37
5.11 Randomization 37
6 Risk Analysis 38
6.1 Risks 38
6.2 Actions to Minimize Increased Risks 39
7 AE Reporting 40
7.1 AE Definitions 40
7.1.1 Adverse Event 40
7.1.2 Anticipated (Expected) Adverse Events 40
7.1.3 Unanticipated (Unexpected) Adverse Events: 40
7.1.4 Serious Adverse Event 41
7.2 Study Termination or Suspension 41
8 Investigator Responsibilities 42

8.1 Investigator Qualifications 42
8.2 IRB\REB Approval 42
8.3 Protocol Adherence 42
8.3.1 Review of Source Documents 42
8.3.2 Record of Investigational Drug Inventory 42
8.3.3 Data Recording and Record Retention 42
8.3.4 Notification Reporting 43
9 Study Data Reporting and Processing 44
9.1 Study Data Collection 44
9.2 Site Data Monitoring and Quality Control 44
9.2.1 Subject Coding 45
9.3 Data Processing and Quality Control 45
9.3.1 Data Cleaning 45
9.3.2 Data Entry 45
9.3.3 Data Editing 45
9.3.4 Data Update 45
9.3.5 Final Data Quality Analyses 46
9.3.6 Data Form Inventory 46
9.3.7 Data Back-up 46
9.3.8 Report Generation and Summary Statistics 46
9.4 Confidentiality and Protection of Study Files 46
10 Study Management 47
10.1 Operations Committee 47
10.2 AOSpine North America Methods Core 47
10.3 Monitoring 47
10.3.1 Pre-Investigation Visits (Site Evaluation Visit) 47
10.3.2 Periodic Visits 47
10.3.3 Record of On-Site Visits 48
10.4 Direct Access to Source Documentation 48
11 Document Control 49
11.1 Protocol 49

11.2 Protocol Amendments 49
11.3 Protocol Deviations 49
11.4 Protocol Violations 49
11.5 Record Retention 49
12 References 50
13 Appendices 52
13.1 Appendix A: Riluzole Drug Insert 52
13.2 Appendix B: Scales and Questionnaires 52

1 Investigator Statement

SPONSOR: AOSpine North America

Efficacy of Riluzole in Patients with Cervical Spondylotic Myelopathy Undergoing Surgical Treatment

A Randomized, Double-Blind, Placebo-Controlled, Multi-Center Study

Protocol Number: SPN-10-001

Version 2.0: February 25, 2013

I, the Investigator of Record, agree to conduct this study in full accordance with the provisions of this protocol and in accordance with applicable regulations and conditions required by the Institutional Review Board/Research Ethics Board (IRB\REB). I agree to maintain all study documentation for a minimum of two years after the study has been completed. Publication of the results of this study will be governed by the conditions stipulated in the Site Agreement. I agree to supervise use of the study product at my institution and ensure the informed consent is obtained prior to subject enrollment.

I have read and understand the information in this protocol and will ensure that all associates, colleagues and employees assisting in the conduct of the study are informed of the obligations incurred by their participation.

____

Name of Site Principal Investigator

___________________________________ ___________________________________

Signature of Site Principal Investigator Date

2 Synopsis

**Official Title**	Efficacy of Riluzole in Patients with Cervical Spondylotic Myelopathy Undergoing Surgical Treatment. A Randomized, Double-Blind, Placebo-Controlled Multi-Center Study
**Short Title**	Efficacy of Riluzole in Surgical Treatment for Cervical Spondylotic Myelopathy (CSM-Protect)
**Sponsor**	AOSpine North America<br>T +1 610 251-9010<br>F +1 610 695-2433<br>1700 Russell Road<br>P.O. Box 1755<br>Paoli, PA 19301, USA
**Purpose**	Riluzole is a potent sodium/glutamate antagonist. It is FDA-approved for the treatment of amyotrophic lateral sclerosis. The purpose of this study is to evaluate if sodium-glutamate antagonist riluzole improves neurological outcomes in patients with cervical spondylotic myelopathy undergoing surgical treatment.
**Condition**	Cervical spondylotic myelopathy
**Intervention**	Drug: riluzole (Rilutek®) 50mg bid<br>Procedure: decompressive/reconstructive cervical spine surgery
**Phase**	III
**Study Type**	Interventional
**Study Design**	Allocation: Randomized<br>Endpoint classification: Efficacy/Safety<br>Model: Parallel assignment<br>Masking: Double blinded<br>Primary purpose: Treatment
**Principal Investigator**	Michael Fehlings MD, PhD, FRCSC<br>Professor of Neurosurgery<br>University of Toronto<br>399 Bathurst St., Suite 4WW-449<br>Toronto Ontario M5T 2S8, Canada<br>Email michael.fehlings@uhn.on.ca
**Co-Principal and Coordinating Investigator**	Branko Kopjar, MD, PhD<br>Associate Professor<br>University of Washington<br>4333 Brooklyn Ave NE<br>Suite 1400/#315<br>Box # 359455

	Seattle, WA 98195, USA<br>Email . brankok@u.washington.edu
**Primary Endpoint**	• Absolute change in mJOA score from admission to 180 days
**Secondary Efficacy Endpoints**	• Absolute change in Nurick score from admission to 180 days<br>• Absolute change in SF-36v2.0™ PCS from baseline to 180 days<br>• Absolute change in Neck Disability Index (NDI) from baseline to 180 days<br>• Absolute change in Cervical Pain Numeric Rating Scale from baseline to 180 days<br>• Absolute change in EQ-5D utility score from baseline to 180 days<br>• Absolute change in ASIA Motor score from baseline to 180 days<br>• Absolute change in ASIA Sensory score from baseline to 180 day<br>• Absolute change in Grip score from baseline to 180 days<br>• Rate of neurological complications
**Estimated Enrollment**	270 subjects randomized 1:1 to riluzole and placebo
**Study Start Date**	April 1, 2011
**Estimated Study Completion**	December 31, 2013
**Arms**	**Investigational**: 50 mg riluzole every 12 hours for 14 days pre and 28 days post decompressive cervical spine surgery<br>**Control**: Placebo and decompressive cervical spine surgery
**Eligibility Inclusion Criteria**	• Signed informed consent<br>• Age between 18 and 80 years<br>• Diagnosis of symptomatic cervical spondylotic myelopathy defined as a combination of:<br>(1) one or more of the following symptoms:<br>o Numb hands<br>o Clumsy hands<br>o Impairment of gait<br>o Bilateral arm paresthesiae<br>o L'hermitte's phenomenon<br>o Weakness<br>And,<br>(2) one or more of the following signs:<br>o Corticospinal distribution motor deficits<br>o Atrophy of hand intrinsic muscles<br>o Hyperreflexia<br>o Positive Hoffman sign<br>o Upgoing plantar responses<br>o Lower limb spasticity<br>o Broad based, unstable gait

	And,<br>(3) MRI evidence of cervical spondylotic myelopathy<br>• Scheduled for an elective surgery for cervical spondylotic myelopathy<br>• mJOA score ≥8 and ≤14 at screening<br>• Women must be:<br>○ Postmenopausal defined as amenorrhea for at least 2 years.<br>○ Surgically sterile, (have had a hysterectomy or bilateral oophorectomy, tubal ligation, or otherwise be incapable of pregnancy)<br>○ Abstinent (at the discretion of the investigator)<br>○ Having other congenital or medical condition that prevents subject from becoming pregnant<br>○ If sexually active, be practicing an effective method of birth control such as hormonal prescription oral contraceptives, progesterone implants or injections, intrauterine device (IUD), or male partner with a vasectomy. A double-barrier method such as condoms, diaphragms or cervical caps with spermicidal foam, cream or gel may be used as a birth control method.<br>○ Women of childbearing potential must have a negative serum β-human chorionic gonadotropin (β-hCG) pregnancy test or a negative urine pregnancy test at screening before the first dose of study drug is received.
**Eligibility Exclusion Criteria**	• Previous surgery for CSM<br>• Concomitant symptomatic lumbar stenosis<br>• CSM symptoms due to cervical trauma (at the discretion of the investigator)<br>• Hypersensitivity to riluzole or any of its components<br>• Neutropenia measured as absolute neutrophil count (ANC) measured in cells per microliter of blood of < 1500 at screening visit<br>• Creatinine level of > 1.2 milligrams (mg) per deciliter (dl) in males or > 1.1 milligrams per deciliter in females at screening visit Liver enzymes (ALT or AST) 3x higher than normal values at screening visit.<br>• Liver enzymes (ALT or AST) 3x higher than normal values at screening visit.<br>• Subject will be using any of the following medications which are classified as CYP1A2 inhibitors or inducers*during the course of the drug regimen:<br>•<br>Inhibitors:<br>• Ciprofloxacin<br>• Enoxacin<br>• Fluvoxamine

	• Methoxsalen<br>• Mexiletine<br>• Oral contraceptives<br>• Phenylpropanolamine<br>• Thiabendazole<br>• Zileuton<br>Inducers:<br>• Montelukast<br>• Phenytoin<br>*Note: no washout period required; if these medications are discontinued, subjects are eligible to be enrolled in the trial.<br>• Systemic infection such as AIDS, HIV, and active hepatitis<br>• Active malignancy defined as history of invasive malignancy, except if the patient has received treatment and displayed no clinical signs and symptoms for at least five years<br>• Recent history (less than 3 years) of chemical substance dependency or significant psychosocial disturbance that may impact the outcome or study participation<br>• Breastfeeding at screening visit and plan to continue during the course of the study drug<br>• Unlikely to comply with the follow-up evaluation schedule<br>• Unlikely to comply with investigational drug regime<br>• Participation in a clinical trial of another investigational drug or device within the past 30 days<br>• Is a prisoner<br>• Unable to converse, read or write English at elementary school level
**Investigational Sites**	AOSpine North America Research Network
**Health Authority**	USA: Institutional Review Boards at participating sites<br>Canada: Health Canada

3 Background Information

3.1 Justification for Investigation

CSM (cervical spondylotic myelopathy) is the most common cause of spinal cord impairment worldwide. The pathophysiology of CSM involves static and dynamic compression of spinal cord which triggers ischemia and secondary cell death by a variety of mechanisms, which prominently include sodium influx and glutamatergic excitotoxicity. While there is emerging evidence from the recently completed AOSpineNA prospective study that surgical decompression is an effective treatment for CSM, it is clear that many patients have substantial residual neurological impairment. Moreover, while surgery is relatively safe, approximately 3% of patients sustain a neurological complication, of which C5 root palsy is the most common adverse outcome. Given this background and compelling evidence from preclinical models of nontraumatic and traumatic spinal cord injury (SCI), there is a strong rationale to consider the potential benefit of adding a neuroprotective drug which targets sodium/glutamate excitotoxicity to the treatment of patients with CSM undergoing surgical decompression. Riluzole, a potent sodium/glutamate antagonist has been widely studied in many models of neurotrauma and neurodegenerative disease. This drug is FDA-approved for the treatment of amyotrophic lateral sclerosis, which has some similar clinical features to CSM. Moreover, riluzole is currently under investigation for traumatic SCI. Given this background, there is a strong rationale to consider studying the potential neurological benefits of riluzole as an adjunctive treatment to surgical decompression in patients with CSM.

3.2 Riluzole

Riluzole tablets, a capsule-shaped, white, film-coated tablet for oral administration containing 50 mg of riluzole. Chemically it is 2-amino-6-(trifluoromethoxy)benzothiazole. Its molecular formula is $C_8H_5F_3N_2OS$ and its molecular weight is 234.2. Its structural formula is as follows:

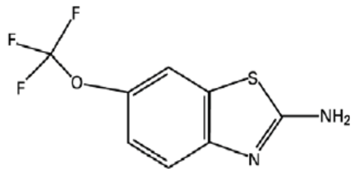

Riluzole, a benzothiazole anticonvulsant Na+ channel blocker, has been shown in two randomized controlled trials to promote increased survival and attenuate neurological dysfunction in patients with amyotrophic lateral sclerosis (ALS), a progressive neurodegenerative disorder characterized by motoneuron and corticospinal tract degeneration (Lacomblez et al., 1996,Hugon, 1996, Bensimon et al., 1994b). A recent Cochrane review has confirmed that the evidence for safety and efficacy in this clinical scenario is persuasive (Miller et al., 2007).

Given the data in the setting of ALS, it is not surprising that riluzole has been examined in other disease models characterized by sodium and glutamate toxicity. Several studies from a number of independent laboratories, in various species of animals, have shown that riluzole is neuroprotective and promotes functional neurological recovery in models of brain and spinal cord ischemic and traumatic injury (Heurteaux et al., 2006, Ates et al., 2007, Lang-Lazdunski et al., 1999, Schwartz and Fehlings, 2001). Other authors have found that the effects of riluzole are synergistic with the effects of methylprednisolone, which is the only drug used in routine clinical practices to attenuate secondary injury effects after SCI (Mu et al., 2000). In a recent study of prolonged administration of riluzole in Huntington's disease, no benefit was found in slowing disease progression, but riluzole was well-tolerated. Adverse effects were virtually similar in 357 subjects treated with riluzole, compared to 180 placebo subjects. Thirteen subjects had elevated liver enzymes and lead to five subjects discontinuing treatment. (Landwehrmeyer et al., 2007).

Riluzole exerts neuroprotective properties in the injured cord following systemic administration by sparing gray and white matter in rostro-caudal regions surrounding the injury epicenter (Schwartz and Fehlings, 2001). In addition to its ability to antagonize Na+ channels, riluzole is also known to inhibit presynaptic Ca2+-dependent glutamate release (Wang et al., 2004). However, given the lack of synaptic connections within white matter, the axon sparing property attributed to riluzole can likely be assigned to its ability to decrease the levels of [Na+]i and intracellular calcium [Ca2+]i.

The use of riluzole as a therapy for SCI is potentially feasible, as it has already received approval from the Food and Drug Administration (FDA) for treatment of amyotrophic lateral sclerosis (ALS)(Miller et al., 2007) at a dose of 100 mg/day. Notably, riluzole is without potent neurotoxic and cardiotoxic adverse effects (Bensimon et al., 1994a), although potential hepatotoxicity has been noted (Bensimon and Doble, 2004). While riluzole is administered for the lifetime of the patient with ALS, it would appear that the duration of therapy in the setting of spinal cord injury would not need to exceed 14 days, based on preclinical animal models and given the anticipated duration of sodium and glutamate mediated secondary injury (Schwartz and Fehlings, 2001,Park et al., 2004).

3.2.1 Pharmacology of Riluzole

In human studies, riluzole has been administered orally in a dose of 50 mg BID. The half-life of riluzole is 12 hours. Most drugs reach steady rate plasma concentrations in 4-5 half lives and the same is assumed for riluzole. Riluzole is highly bound to plasma proteins – 97.5%, like phenytoin. In subjects taking other drugs that bind to plasma proteins the binding sites compete and presumably result in more free riluzole in the plasma with greater drug activity. Riluzole is metabolized in the liver by an enzyme of the cytochrome P (CYP) 450 family. There are multiple CYP genes; however, most of the drugs metabolizing enzymes are in the CYP 1, 2 & 3 families. Riluzole is specifically metabolized by a member of the CYP 1A2 subfamily whose substrates include acetaminophen, caffeine and warfarin. Other drugs that are metabolized by CYP 1A2 are Tacrine (Cognex), Omeprazole (Prilosec) and quiniolone antibiotics and theophylline. Co-administration of riluzole and these drugs can increase riluzole blood concentration.

3.2.2 Regulatory Status

Riluzole is approved by FDA and Health Canada for treatment of amyotrophic lateral sclerosis. It is not approved by the FDA or by Health Canada for treating patients undergoing cervical surgical decompression for cervical spondylotic myelopathy.

The IND regulations [21 CFR 312.2(b)] state that clinical investigation of a drug product that is lawfully marketed in the United States is exempt from the requirements for an IND if all of the following apply: 1. The investigation is not intended to be reported to FDA as a well-controlled study in support of a new indication for use, nor intended to be used to support any other significant change in the labeling for the drug.; 2. The investigation is not intended to support a significant change in the advertising for a prescription drug product.; 3. The investigation does not involve a change in route of administration, dosage level, or patient population, or other factor that significantly increases the risks (or decreases the acceptability of risks) associated with use of the drug product.; 4. The investigation is conducted in compliance with the requirements for institutional review (21 CFR Part 56) and informed consent (21 CFR Part 50); 5. The investigation is conducted in compliance with the requirements of 21 CFR 312.7, i.e., the drug may not be represented as safe or effective for the purposes for which it is under investigation, nor may it be commercially distributed or sold. In addition, 21 CFR 312.2(b)(5)states a clinical investigation involving the use of a placebo is exempt from the IND requirements if the investigation does not otherwise require submission of an IND.

The FDA has been contacted by the Sponsor requesting clarification as to whether the trial meets the above requirements for IND exemption. The Sponsor has received a letter from the FDA stating that they acknowledge receipt of the IND Application and after review, it has been determined that the trial meets all of the requirements for exemption and therefore and IND is not required to conduct the investigation in accordance with 21 CFR 312.2(b)(4)

Health Canada regulates investigator-driven research in the same way as industry-sponsored research. The “No Objection Letter” (equivalent to US FDA IND approval) will be obtained from Health Canada prior to beginning the study at Canadian sites.

3.3 Study Scope and Duration

This is a multi-center, prospective, double-blinded, randomized placebo-controlled clinical trial to evaluate if sodium-glutamate antagonist riluzole in a dose of 50mg BID 14 days before the surgery and continuing for 28 days after the surgery is superior to placebo in patients with moderate and severe cervical spondylotic myelopathy undergoing surgical decompression.

The study will involve up to 16 investigational sites and enroll 270 evaluable subjects. A randomization ratio of one riluzole subject to one placebo control subject (1:1) will be utilized. The sample size may change during the interim analysis due to adaptive statistical design.

Patients scheduled for elective surgical decompression due to cervical spondylotic myelopathy will be screened for inclusion in the study. If the patient meets all of the eligibility criteria, he/she will be enrolled and randomized to either the riluzole or placebo controlled group. The randomization will occur 14-17 days prior to surgery. Subjects will begin receiving study medications 14 days prior to the planned surgery date and continue receiving it for 28 days following the surgery. The riluzole group will receive riluzole in a dose of 50 mg every 12 hours. The control group will receive placebo capsules that are the same color, size, taste and

consistency as riluzole capsules but will not have the active ingredient. Subjects, investigators, and study research personnel will be blinded to the randomization code. The study Contract Research Organization (CRO) will maintain randomization and blinding. Treatment allocation will be un-blinded for the purpose of interim analysis. The results of the interim analysis and the un-blinded data will not be shared with investigators, subjects or those involved in the execution of the study.

Subjects will be evaluated according to the schedule set forth in this clinical protocol. The schedule includes an evaluation at 14 days before the surgery (- 14 days), admission, surgery (Day 0), post-surgery, a postoperative discharge exam, 35 days, 180 days and 365 days. The 365-day follow-up will be performed until the last subject enrolled and still active in the study (i.e. not withdrawn) has completed the 180-day follow-up. The enrollment period is expected to be approximately 24 months and subjects will be followed for 6 months for the evaluation of the primary efficacy endpoint. The study duration is approximately 30 months.

4 Design of the Study

4.1 Objectives of the Study

The primary objective of this study is to evaluate whether sodium-glutamate antagonist riluzole in a dose of 50mg BID 14 days prior to the surgery and 28 days following the surgery is superior to placebo in achieving better neurological outcomes in patients undergoing surgical decompression for moderate or severe cervical spondylotic myelopathy.

Secondary objectives are to evaluate differences between the investigational and the control group as measured by pain, functional and quality of life outcomes, health utilities, and adverse events.

4.1.1 Primary Endpoint

- Absolute change in mJOA from admission to 180 days

4.1.2 Secondary Efficacy Endpoints

- Absolute change in Nurick score from admission to 180 days
- Absolute change in SF-36v2.0™ PCS from admission to 180 days
- Absolute change in Neck Disability Index (NDI) from admission to 180 days
- Absolute change in Cervical Pain Numeric Rating Scale from admission to 180 days
- Absolute change in EQ-5D utility score from admission to 180 days
- Absolute change in ASIA Motor score from admission to 180 days
- Absolute change in ASIA Sensory score from admission to 180 days
- Absolute change in Grip Strength from admission to 180 days
- Rate of neurological complications

4.1.3 Other Endpoints

- Absolute change in mJOA score from admission to 35 days and 365 days
- Absolute change in Nurick score from admission to 35 days and 365 days
- Absolute change in SF-36v2.0™ PCS score from admission to 35 days and 365 days
- Absolute change in NDI score from admission to 35 days and 365 days
- Absolute change in SF-36v2.0™ MCS score from admission to 35 days and 365 days
- Absolute change in SF-36v2.0™ eight dimensions from admission to 35 days and 365 days
- Absolute change in EQ-5D from admission to 35 days and 365 days
- Absolute change in ASIA Motor Score from admission to 35 days and 365 days
- Absolute change in ASIA Sensory Score from admission to 35 days and 365 days
- Absolute change in Grip Strength from admission to 35 days and 365 days
- Absolute change in Cervical Pain NRS from admission to 35 days and 365 days
- Absolute change in Bazaz Dysphagia Score from admission to 35 days and 365 days
- Systemic (non-neurological) complications

- Changes in all outcome parameters (mJOA, Nurick, SF36v2, NDI, EQ-5D, ASIA Motor Score, ASIA Sensory Score, Grip Strength, Cervical Pain NRS and, Bazaz Dysphagia Score) between -14 days and the pre-operative visit
- Changes in outcome parameters over follow-up time (-14, admission, 35, 180 and, 365 days).

4.1.4 Safety Evaluation

Incidence of adverse events (AE) will be summarized by body system and preferred term. All AEs will be listed and abnormal lab values reviewed. AEs will be adjudicated for relationship to the investigational drug.

4.2 Selection of Investigators

Investigators will be selected among the AOSpine North America Research Network associated research centers. All AOSpine North America Research Network investigators are registered orthopedic and neurological surgeons who meet the credentials at their institution for performing spine surgical procedures. The surgical procedures used in this study are considered standard of care. Selected investigational site will provide evidence of qualification for conducting clinical research to Sponsor /CRO.

4.3 Inclusion Criteria

- Signed informed consent
- Age between 18 and 80 years
- Diagnosis of symptomatic cervical spondylotic myelopathy defined as a combination of:
  (1) one or more of the following symptoms:
    - Numb hands
    - Clumsy hands
    - Impairment of gait
    - Bilateral arm paresthesiae
    - l'Hermitte's phenomena
    - Weakness

  And,
  (2) one or more of the following signs:
    - Corticospinal distribution motor deficits
    - Atrophy of hand intrinsic muscles
    - Hyperreflexia
    - Positive Hoffman sign
    - Upgoing plantar responses
    - Lower limb spasticity
    - Broad based, unstable gait

  And,
  (3) MRI evidence of cervical spondylotic myelopathy
- Scheduled for an elective surgery for cervical spondylotic myelopathy
- mJOA score ≥8 and ≤14 at screening

- Women must be:
  - Postmenopausal defined as amenorrhea for at least 2 years.
  - Surgically sterile, (have had a hysterectomy or bilateral oophorectomy, tubal ligation, or otherwise be incapable of pregnancy)
  - Abstinent (at the discretion of the investigator)
  - Having other congenital or medical condition that prevents subject from becoming pregnant
  - If sexually active, be practicing an effective method of birth control such as hormonal prescription oral contraceptives, progesterone implants or injections, intrauterine device (IUD), or male partner with a vasectomy. A double-barrier method such as condoms, diaphragms or cervical caps with spermicidal foam, cream or gel may be used as a birth control method.
  - Women of childbearing potential must have a negative serum β-human chorionic gonadotropin (β-hCG) pregnancy test or a negative urine pregnancy test at screening before the first dose of study drug is received.

4.4 Exclusion Criteria

- Previous surgery for CSM
- Concomitant symptomatic lumbar stenosis
- CSM symptoms due to cervical trauma (at the discretion of the investigator)
- Hypersensitivity to riluzole or any of its components
- Neutropenia measured as absolute neutrophil count (ANC) measured in cells per microliter of blood of < 1500 at screening visit
- Creatinine level of > 1.2 milligrams (mg) per deciliter (dl) in males or > 1.1 milligrams per deciliter in females at screening visit
- Liver enzymes (ALT or AST) 3x higher than normal values at screening visit.
- Subject will be using any of the following medications which are classified as CYP1A2 inhibitors or inducers*during the course of the drug regimen:
  Inhibitors:
  - Ciprofloxacin
  - Enoxacin
  - Fluvoxamine
  - Methoxsalen
  - Mexiletine
  - Oral contraceptives
  - Phenylpropanolamine
  - Thiabendazole
  - Zileuton

  Inducers:
  - Montelukast
  - Phenytoin

*Note: no washout period required; if these medications are discontinued, subjects are eligible to be enrolled in the trial

- Systemic infection such as AIDS, HIV, and active hepatitis

- Active malignancy defined as history of invasive malignancy, except if the patient has received treatment and displayed no clinical signs and symptoms for at least five years
- Recent history (less than 3 years) of chemical substance dependency or significant psychosocial disturbance that may impact the outcome or study participation
- Breastfeed at screening visit and plan to continue during the course of the study drug
- Unlikely to comply with the follow-up evaluation schedule
- Unlikely to comply with investigational drug regime
- Participation in a clinical trial of another investigational drug or device within the past 30 days
- Is a prisoner
- Unable to converse, read or write English at elementary school level

4.5 Study Procedures

4.5.1 Study Schematic

The schedule of visits and data to be collected at each visit is shown in Table 1. All visits must be performed in clinic. Data collection by phone and/or mail is not permitted.

Table 1 Schedule of study activities

	Screening	Enrollment (15--21 days before the surgery)	Admission	Procedure (Day 0)	Pre-discharge	35 days ± 5 days	180 days ± 30 days	365 days ±30 days	Unscheduled Visit
Informed Consent	X								
HIPAA Authorization	X								
Inclusion/exclusion	X								
Pregnancy Test (If applicable)	X								
Demographics	X								
Socio-economic		X							
Health behavior		X	X			X	X	X	
Medical History		X							
Vitals		X	X		X	X			
Clinical lab	X		X			X			
Myelopathy History		X							
Operative data				X					
mJOA	X	X††	X			X	X	X	
Nurick score		X	X			X	X	X	
NDI		X	X			X	X	X	
Pain NRS		X	X			X	X	X	
SF-36v2.0™		X	X			X	X	X	

	Screening	Enrollment (15--21 days before the surgery)	Admission	Procedure (Day 0)	Pre-discharge	35 days ± 5 days	180 days ± 30 days	365 days ±30 days	Unscheduled Visit
**EQ-5D**		X	X			X	X	X	
**ASIA (Complete)**		X	X			X	X	X	
**Grip Strength**		X	X			X	X	X	
**Bazaz Scale**		X	X			X	X	X	
**Adverse Events**			X	X	X	X	X	X	X
**Concomitant therapy**		X	X		X	X	X	X	X
**Randomization**		X							
**Study Medication Dispensing**		X	X						
**Medication Compliance Diary**			X			X			

†† mJOA needs to be repeated if older than 21 days or if patient's clinical manifestation has significantly worsened in investigator's opinion since the screening visit

4.5.2 Study Time Line

All follow-up windows are based on “Day 0” which for this study is the day of cervical spondylotic myelopathy surgery.

4.5.2.1 Pre-screening

Patients receiving care at the investigative sites will be pre-screened as potential subjects for the study. In order to do so, only the existing information obtained per standard routine medical procedures will be used. No study-specific screening procedures, activities or questionnaires will be performed during the pre-screening. Patients who fail pre-screening will not be recorded on any log or form.

4.5.2.2 Visit 1: Screening[1](15 or more days prior to surgery)

The first visit is the screening visit.

Patients considered potential candidates for the study based on pre-screening will sign an Internal Review Board (IRB)/Research Ethics Board (REB) approved informed consent form prior to participating in any study activities

- Obtain signed informed consent, place it in the patient file, make a copy and give it to the patient;
- Obtain signature on the HIPAA Authorization Form or equivalent Canadian health information release form;
- Assign a Subject Investigational Code (SIC);
- Verify that the patient meets all inclusion/exclusion criteria and perform the following:
    - Demographics
    - mJOA instrument (clinician administered) and calculation of the score
    - Clinical laboratory blood tests (liver function, creatinine, absolute neutrophils count). Test results obtained within 30 days before the screening are acceptable to this study
    - Pregnancy test (if applicable)
- Check Screening source worksheets for completeness
- Verify data and enter into eCRF within 14 calendar days of collection

4.5.2.3 Visit 2: Enrollment (15-21 days prior to surgery)

This visit occurs 15-21 days before the surgery and prior to the first dose of study medication. This visit can be performed on the same day as the screening visit or on two separate days. If performed on two separate days, the Investigator and the Study Coordinator should ensure that:

- The screening data are current

[1] Visit 1 and Visit 2 can be combined into one physical visit

- The patient's screening mJOA test was performed in less than 21 days prior to surgery. If not, repeat the mJOA.
- The patient's clinical manifestation has not significantly worsened (investigator's opinion) since the Visit 1. If yes, repeat the mJOA.

If the patient fails inclusion/exclusion criteria, the patient should not be enrolled to the study by the Investigator and the subject will be handled as a screen failure, the reason for the screen failure will be recorded on the screening source worksheet, data shall be entered into eCRF with in 14 calendar days of data collection.

The following procedures are performed during the visit:

- Obtain socio-economic, health behavior, medical history, vitals, and information about concomitant medications
- Investigator:
    - Perform and record mJOA, Nurick Score, Grip Strength and complete ASIA examination
- Subject completes questionnaires:
    - NDI, Pain NRS, SF-36v2™, EQ-5D, Bazaz
- Review and verify all data collected to confirm the subject meets all study inclusion/exclusion criteria.
- **Enroll subject**
- Perform randomization procedure and enter randomization code into the Randomization Log
- Update information in the Screening Form
- Provide subject with *Bottle#1* of study medication and record on the Study Medication Dispensing CRF
- Provide subject with a Medication Compliance Diary to fill out the information about the treatment dates and times
- Teach subject how to take the medication and fill out the Medication Compliance Diary
- Inform subject to bring the Medication Compliance Diary and any unused medication with its container to the next visit (admission)
- Log the subject's drug accountability information into the Site Drug Accountability Log
- Advise subject not to take medications in the exclusion list
- Check source worksheets for completeness
- Verify data and enter into eCRF within 14 calendar days of collection

The first treatment dose should be administered in the morning of the 14th day before the surgery so that the surgery falls on the 15th day. For example, if the surgery is scheduled on Monday, the first dose of treatment medication will be administered on Monday morning two weeks before the surgery.

If the visit has occurred prior to the 14th day before the surgery, the site research coordinator will call the subject on the day -15 to ensure that the first dose of the medication will be administered as scheduled.

4.5.2.4 Visit 3: Admission (pre-surgery)

This visit occurs when the patient is admitted to the hospital for surgery. During the hospital stay the subject should receive scheduled study medication. Study coordinator should coordinate with the hospital pharmacy to assure that the subject receives the study medication during the hospital stay.

The following activities should be performed at admission:

- Record date and time of hospital admission
- Check and record AEs/SAEs
- Check and record concomitant therapy
- Obtain and record vital signs
- Obtain blood sample and order clinical lab tests
- Collect the medications and the Bottle #1 dispensed at enrollment visit.
- Count and record the remaining medication. The remaining medication and its container will be counted and compared with the data from Subject's Medication Compliance Diary. Record study medication compliance on the Medication Compliance CRF.
- Ensure the subject receives study medication during the hospital stay, and record in the Subject's Medication Compliance Diary. Bottle #2 should be dispensed on the surgery day (Day 0)
- Log the subject's drug accountability information into the Site Drug Accountability Log
- Investigator:
    - Obtain and record mJOA, Nurick, Grip Strength
    - Perform and record ASIA (complete)
- Subject:
    - Fill-out entire Subject pre-surgical source worksheet with data on health behavior, NDI, SF36v2.0, EQ-5D, Pain NRS, Bazaz
- Check source worksheets for completeness
- Verify data and enter into eCRF within 14 calendar days of collection

4.5.2.5 Visit4: Procedure

- Record operative data into source worksheets
- Obtain and record intra-operative and immediate postoperative AE/SAE
- Check source worksheets for completeness
- Verify data and enter into eCRF within 14 calendar days of collection

4.5.2.6 Visit 5: Pre-discharge

- Obtain and record vitals
- Obtain and record post-operative AE/SAEs
- Check and record concomitant therapy

- Provide subject with the remainder supply of study medication and record on the Medication Dispensing CRF
- Advise the subject the date when to stop taking trial medication (i.e. the 28th day after surgery is the last day to take the trial medication).
- Schedule appointment for the next visit (35 days after the date of surgery)
- Record time and date of hospital discharge
- Check source worksheets for completeness
- Verify data and enter into the eCRF within 14 calendar days of collection

4.5.2.7 Visit 6: 35 days ± 5 days

This is the first scheduled follow-up visit. The visit should occur at earliest 30 days from the date of surgery to allow subject to complete the 28 days of post-surgical treatment. The following activities should be performed during the visit.

- Subject returns bottle #2 with any remaining medication along with the Subject's Medication Compliance Diary. The remaining medication will be compared to the Subject's Medication Compliance Diary.
- Obtain and record concomitant therapy
- Obtain and record AE/SAE
- Obtain and record vitals
- Investigator performs and records:
  - ASIA (complete)
  - mJOA
  - Nurick
  - Grip Strength
- Obtain blood sample and order clinical lab tests
- Subject completes questionnaires for:
  - Health behavior
  - NDI
  - Pain NRS
  - EQ-5D
  - SF-36v2™
  - Bazaz
- Record study medication compliance in Medication Compliance CRF
- Log the subject's drug accountability information into the Site Drug Accountability Log
- Schedule next study visit (180 days post-surgery)
- Check Source Worksheets for completeness
- Verify data and enter into eCRF within 14 calendar days of collection

4.5.2.8 Visit 7: 180 days ±30 days

This is the main endpoint study visit. The following activities should be performed during the visit.

- Obtain and record concomitant therapy
- Obtain and record AE/SAE

- Investigator performs and records:
  - ASIA (complete)
  - mJOA
  - Nurick
  - Grip Strength
- Subject completes questionnaires for:
  - Health behavior
  - NDI
  - Pain NRS
  - EQ-5D
  - SF-36v2™
  - Bazaz
- Schedule next study visit (365 days post-surgery)
- Check Source Worksheets for completeness
- Verify data and enter into eCRF within 14 calendar days of collection

4.5.2.9 Visit 8: 365 days ±30 days

The following activities should be performed during the visit.

- Obtain and record concomitant therapy
- Obtain and record AE/SAE
- Investigator performs and records:
  - ASIA (complete)
  - mJOA
  - Nurick
  - Grip Strength
- Subject completes questionnaires for:
  - Health behavior
  - NDI
  - Pain NRS
  - EQ-5D
  - SF-36v2™
  - Bazaz
- Check Source Worksheets for completeness
- Verify data and enter into eCRF within 14 calendar days of collection

4.5.2.10 Unscheduled Visit

- Obtain and record concomitant therapy
- Obtain and record AE/SAE
- Check Source Worksheets for completeness
- Verify data and enter into eCRF within 14 calendar days of collection

4.5.3 Enrollment and Randomization

Patient enrollment occurs at the time of randomization. If a patient is consented, screened, and, for any reason, does not get randomized, the patient will not be considered enrolled into the study.

Patient randomization occurs at Visit 2 (Enrollment), 15-21 days prior to the surgery. The randomization will occur by opening the lowest sequential number of the sealed randomization envelops. Inside the envelope there will be a unique patient randomization number. The study coordinator will match the unique patient randomization number with the number on the medication container. The randomization will be recorded in the Randomization Log.

4.5.4 Study Drug Procedures

4.5.4.1 Dispensing the study drug

Each study site will be supplied with the study drug. All study drug containers will have identical appearance. Each container will be labeled with a unique number that will match the number in the randomization envelope. Bottle#1 will contain 17-day supply (i.e. 34 capsules) of the study medication and Bottle#2 will contain 31-days supply (i.e. 62 capsules).

Authorized site research staff will select the study drug package to dispense to the subject by matching the container label to the unique randomization number identified in the randomization envelope. Randomization will be recorded in the Randomization Log.

Study Subjects will be instructed:

- To take one capsule every 12 hours.
- Study medication should be taken at least one hour before, or 2 hours after a meal to avoid food-related decrease in bioavailability.
- If a dose is missed, take the next capsule as originally planned.
- To store the study drug at the temperature between 20°-25°C (68°-77°F) protected from bright light.

4.5.4.2 Breaking the Randomization Code

The CRO will be responsible for randomization assignment and will hold a key to un-blind the case assignment in case of emergency. In the event of an emergency the principal investigator at the site will make the decision about the un-blinding of the case. The reason for un-blinding will be recorded and submitted to IRB\REB and Sponsor/CRO.

4.5.4.3 Storage

Investigational product/placebo must be stored in a locked cabinet with limited access. The drug should be stored at the temperature between 20°-25°C (68°-77°F) protected from bright light. A temperature log shall be maintained at the study site.

4.5.4.4 *Study Drug Accountability*

Study drug shipment to sites will be accompanied by a Drug Shipment Form which must be signed and dated upon receipt and faxed back to the sponsor/CRO. This form must be filed in the Regulatory Binder. Drug accountability, per patient, will be performed by investigator staff at the Enrollment, Admission and 35-days visit.

The total study inventory by site and for the study as a whole will be reconciled at the end of the study. Any unused drug inventory will be processed for disposal or return.

Research pharmacist or the Site Coordinator, if Research Pharmacist is not available at the site, responsible for the study will detail the procedures for handling the Study Drug.

4.5.4.5 *Assessment of Patient Compliance*

Compliance will be recorded by the study research coordinator or investigator during the Admission and 35-days follow-up visits and must be documented on the appropriate source document (Medication Compliance section). Subjects must have 80% or greater compliance in each of the medication periods (14-17 days prior to surgery and 28 days after the surgery.)

- As an example, the subject is considered compliant in the period 14 days prior to surgery if he/she has taken at least 23 capsules.
- The subject is considered compliant in the 28 days medication treatment period after the surgery if he/she has taken at least 45 capsules.

Failure to comply will be considered a Protocol Violation and will be recorded on the Protocol Violation Log. Non-compliant subjects will remain in the study and will undergo the same study procedures as compliant subjects.

4.5.5 Surgical Procedure

The decompressive/reconstructive surgery for CSM will be performed per standard of care at the treating institution. The surgical approach, medical devices and bone grafts/bone graft substitutes used will be at the surgeon's discretion.

4.5.6 Post-surgical Rehabilitation and Procedures

Post-treatment procedures will be per the standard of care.

4.5.7 Clinical Laboratory Procedures

Table 2 Clinical lab blood tests

Examination	Screening	Admission	35 days ± 5 days
AST, SGOT	X	X	X
ALT, SGPT	X	X	X
Creatinine (mg/dL)	X	X	X
Red Blood Cell (RBC) Count (x10^6/μL)	X	X	X
White Blood Cell (WBC) Count (x10^6/μL)	X	X	X
Hemoglobin or Hbg (gm/dL)	X	X	X
Hematocrit or Hct (%)	X	X	X
Polymorphonuclear Cells (%)	X	X	X
Immature Polys (%)	X	X	X
Lymphocytes (%)	X	X	X
Monocytes (%)	X	X	X
Eosinophils (%)	X	X	X
Basophils (%)	X	X	X
Platelet Count (x10^3/μL)	X	X	X
Mean Cell Hemoglobin Concentration (gm/dL)	X	X	X
Reticulocyte (%)	X	X	X

4.6 Subjects Completion and Disposition

4.6.1 Screen Failure

Screen failure is defined as a subject who has signed Informed Consent Form but does not meet the inclusion or exclusion criteria. A subject may be considered a screen failure any time prior to randomization. Screen failures will complete the screening source worksheet and the reason for failure will be provided.

4.6.2 Enrolled Subject

A subject is considered to be enrolled when the randomization occurs.

4.6.3 Withdrawn Subject

Reasonable efforts to keep each subject in the study will be made and must be documented by the investigator. A subject will be withdrawn from the study for any of the following reasons:

- Subject voluntarily withdraws consent after the randomization and terminates participation. Such subject will not be replaced.
- The investigator withdraws the subject at any time for safety reasons or non-compliance with the study protocol or procedures. Such subject will not be replaced.

For each case, detailed information will be obtained explaining circumstances leading to the withdrawal. This will be recorded on the Subject Withdrawal Form and in the source document. Study drug assigned to the withdrawn subject shall not be assigned to another subject. The remaining study medication for the withdrawn subject will be returned from subject and/or kept at the site and processed at the end of the study according to the disposal or return instructions. For safety reasons, the subject who withdraws from the study for any reason before completion of the dose regiment and the last scheduled lab test will be provided for safety evaluation purpose. This shall occur within 30 days of the last dose of the Study Drug.

4.6.4 Lost to Follow-up

A subject will be considered lost to follow-up if they do not come for the scheduled study visit and the study personnel is unable to contact the subject. The study personnel must perform and document the following contact attempts prior to declaring a subject to be lost to follow-up: three phone calls with at least 2 days in between each call followed by a certified letter. The first phone call must occur latest 3 business days after subject has failed to show up for the visit.

4.6.5 Completed Subject

A subject will be considered as having completed the study if he/she has completed all assessments through the 365-day visit. Subjects who do not reach 365 days since the surgery at the time when the trial is stopped for enrollment will be considered as completed if they have completed all assessments through the 180-day visit. If the subject has reached 365 days since surgery before the end of study enrollment, but subject missed visit(s) due to being withdrawn, lost to follow-up, or deceased, he/she will not be categorized as having completed the study.

5 Statistical Analysis

Details of the statistical analysis will be included in the Statistical Analysis Plan (SAP), which will be provided as a separate document. This section provides key elements of the statistical approach.

5.1 Primary Efficacy Endpoint

The primary efficacy endpoint in this study is absolute difference in mJOA between 180 days follow-up and baseline.

$$\Delta mJOA_{180\text{-}b} = mJOA_{180\ days} - mJOA_{baseline} \quad (1)$$

5.2 Secondary Efficacy Endpoints

1. Absolute change in Nurick score from admission to 180 days
2. Absolute change in SF-36v2.0™ PCS from admission to 180 days
3. Absolute change in Neck Disability Index (NDI) from admission to 180 days
4. Absolute change in Cervical Pain Numeric Rating Scale from admission to 180 days
5. Absolute change in EQ-5D utility score from admission to 180 days
6. Absolute change in ASIA Motor Score from admission to 180 days
7. Absolute change in ASIA Sensory Score from admission to 180 days
8. Absolute change in Grip Strength from baseline to 180 days
9. Rate of neurological complications

5.3 Other Endpoints

1. Absolute change in mJOA score from admission to 35 days, and 365 days
2. Absolute change in Nurick score from admission to 35 days, and 365 days
3. Absolute change in SF-36v2.0™ PCS score from admission to 35 days, and 365 days
4. Absolute change in NDI score from admission to 35 days, and 365 days
5. Absolute change in SF-36v2.0™ MCS score from baseline to 35 days, and 365 days
6. Absolute change in SF-36v2.0™ eight dimensions from baseline to 35 days, and 365 days
7. Absolute change in EQ-5D from baseline to 35 days, and 365 days
8. Absolute change in ASIA Motor Score from baseline to 35 days, and 365 days
9. Absolute change in ASIA Sensory Score from baseline to 35 days and 365 days
10. Absolute change in Grip Strength from baseline to 35 days, and 365 days
11. Absolute change in Cervical Pain NRS from admission to 35 days, and 365 days
12. Absolute change in Bazaz from admission to 35 days, 180 days, and 365 days
13. Systemic (non-neurological) complications
14. Changes in all outcome parameters (mJOA, Nurick, SF36v2, NDI, EQ-5D, ASIA Motor Score, ASIA Sensory Score, Grip Strength, Cervical Pain NRS, Bazaz) between -14 days and the pre-operative visit

15. Changes in outcome parameters over follow-up time (-14, pre-operative, 35, 180 and 365 days).

5.3.1 Complications and AE

Complications and AE will be recorded according to descriptions provided in Section 6.

5.4 Study success

5.4.1 Main Definition of Success

The study success is defined as follows:

Investigational treatment (riluzole) is superior to placebo. The study success is achieved if one-sided null hypothesis of no superiority of riluzole group in $\Delta mJOA_{180\text{-}b}$ is rejected.

5.4.2 Alternative Definition of Success

The alternative definition of success is if ANY of the following secondary efficacy hypotheses has been rejected.

1. One-sided null hypothesis of no superiority of riluzole in change in ASIA Motor Score between the baseline and the 6 months post-surgery.
2. One-sided null hypothesis of no superiority of riluzole in change in Nurick score between the baseline and the 6 months post-surgery.
3. One-sided null hypothesis of no superiority of riluzole in change in NDI score between the baseline and the 6 months post-surgery.

5.5 Statistical Testing of Primary Endpoint (mJOA)

The appropriate statistical approach is to test a single one-sided null-hypothesis that the difference between the Investigational and the Placebo arm is equal to or less than 0. Rejection of null-hypothesis is consistent with superiority of investigational treatment.

$$H0: \mu_I - \mu_c \leq 0$$

$$H1: \mu_I - \mu_c > 0$$

Where:

μ_I and μ_C are the means of the two independent normal distributions;

μ_I is the mean population value of the $\Delta mJOA_{180\text{-}b}$ in the patients treated with riluzole;

μ_c is the mean population value of the $\Delta mJOA_{180\text{-}b}$ in the patients treated with placebo.

The hypothesis will be tested by one-sided t-test at the alpha level of 0.025. If the test value exceeds the nominal critical point, the H0 will be rejected.

5.6 Statistical Testing of Secondary Endpoints

Three secondary endpoints will be tested for superiority in the primary study success analysis as alternative definition of study success. This testing will be performed only if the primary study success testing fails. The statistical significance will be established by one-sided $\alpha = 0.025$. Appropriate adjustments will be made to account for impact on Type I error using methods described by Hochberg (1988).

5.7 Sample Size Estimate

The required sample size is 270 evaluable subjects, or 135 subjects per study arm. The rationale for the sample size is as follows.

Sample size has been calculated to provide 80% power in testing the primary superiority hypothesis. The estimate has been made by the sample size estimation software package East v5.3 (Cytel, Inc.).

Using the data from the performed CSM study (data on file with the Sponsor), the following empirical estimates have been obtained for the primary outcome parameters.

Endpoint	N	Mean	Standard Deviation
$\Delta mJOA_{180-b}$	146	2.81	2.57

Table 3 Sample size estimate $\Delta mJOA_{180\text{-}b}$

Plan ID	Parameter
Type of the hypothesis	1-Sided
Type I Error (α)	0.025
Power (1 - β)	0.80
Randomization Ratio (Investigational vs. Control)	1:1
Planned Number of Interim Looks	2
Spacing of Looks	65%, 100%
Hypothesis to be Rejected	H0 or H1 (binding)
Boundary Family	Published Function
Boundary to Reject H0	O'Brien-Fleming
Boundary to Reject H1	Gamma (-2)
Difference of Means Assuming H_1	0.9
Standard Deviation (σ)	2.57
Sample Size	270 (135 per arm)

Under the above assumptions, the estimated sample size is 270 evaluable subjects. (135 in the riluzole arm and 135 in the control arm). In order to account for loss of power due to loss of follow-up and possible adjustments for baseline factors we will increase sample size for 10% to **300** *enrolled* subjects.

5.8 Statistical Performance and Simulations

The statistical plan aims at efficient design that allows demonstrating efficacy of the treatment while allowing for early stop for futility if the treatment does not appear to be successful.

Figure 1 shows rejection boundaries for H0 and H1 on the absolute delta scale for the interim and the final analysis. The figure shows that the futility stop is at the value of about 0.35 in favor of treatment. In other words, if the difference between the investigational and the control arms is less than 0.35, the study may stop for futility. At the same time, if the difference is about 1.0 absolute point the study may reach efficacy endpoint.

Figure 1 Rejection boundaries for mJOA

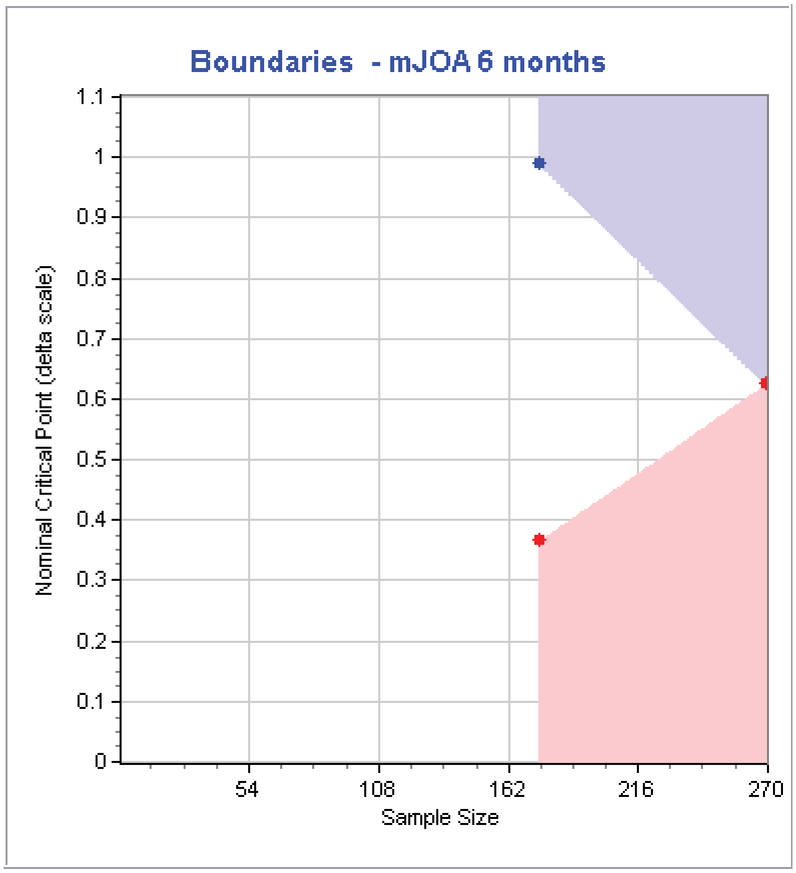

Table4 shows simulation results of the testing under the assumption of no difference between the investigational and control groups. The simulation shows that under those conditions, the H1 will be rejected in 82.5% of the cases at the interim analysis (futility stop) and at 97.64% cases at the final analysis.

Table5 shows simulation results of the testing under the assumption of a treatment effect of the size of 1 absolute point in mJOA, the background for the sample size estimate. The simulation shows that under those conditions, the H0 will be rejected in 40.95% of the cases at the first interim analysis, and in cumulative 80.05% at the last analysis.

Table 4. Simulation results: difference between investigational and control group is 0 points on mJOA scale

Normal Superiority Trials: Two-Sample Test - Difference of Means (Enhanced Simulation)

Plan Details	
1-Sided or 2-Sided Test	1-Sided
Significance Level (α)	0.025
Pow er (1 - β)	0.8
Assigned Fraction (Treatment)	0.5
Planned Number of Looks	2
Spacing of Looks	Unequal
Hypothesis to be Rejected	H0 or H1 (NB)
Boundary Family	SpF (Pub)
Boundary to Reject H0	LD (OF)
Boundary to Reject H1	Gm (-2)
Difference of Means (δ_1)	0.9
Standard Deviation (σ)	2.57
Maximum Sample Size	270

Simulation Boundaries						Latest Simulated Test Stat.	Overall Simulation Results				Total Simulations	
Look #	Samp Size	Boundary H0-	H0+	H1-	H1+		Avg. Information	Avg.Sample Size	# Rejecting H0	# Rejecting H1	Count	%
1	175.26		2.5469		0.9365	-0.5658	6.68	175.00	70	8250	8320	83.20%
2	269.64		1.9896		1.9896		10.33	270.00	166	1514	1680	16.80%
3												
4												
5												
6												
7												
8												
9												
10												
11												
12												
						**Total**	7.29	190.96	236	9764	10000	
						**%**			2.36%	97.64%		

Design Outputs	
Max. Sample Size (Nmax)	270
Max. Information (Imax)	10.2060

Simulation Parameters	
Simulation Scale	Sample Size
Difference of Means	0
Standard Deviation	2.57
Number of Trials	10000
Refresh Every 'n' Trials, n =	1000
Simulation Starting Seed	Clock

Run | Single Step | Reset | Stop

Trial # = 10000
Simulation Seed = 37669
Elapsed Time = 0:00:11

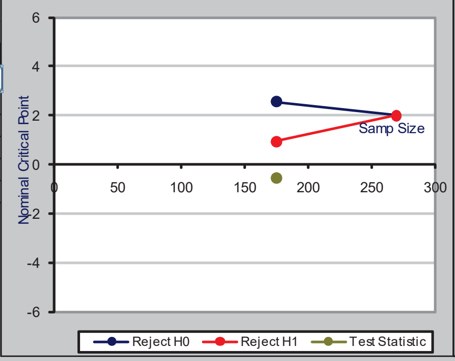

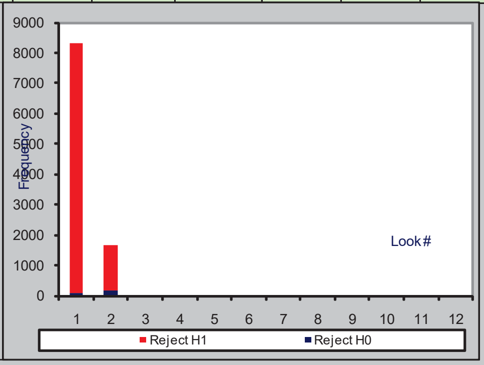

Table 5. Simulation results: difference between investigational and control group is 0.9 absolute points in mJOA

Normal Superiority Trials: Two-Sample Test - Difference of Means (Enhanced Simulation)

Plan Details	
1-Sided or 2-Sided Test	1-Sided
Significance Level (α)	0.025
Pow er (1 - β)	0.8
Assigned Fraction (Treatment)	0.5
Planned Number of Looks	2
Spacing of Looks	Unequal
Hypothesis to be Rejected	H0 or H1 (NB)
Boundary Family	SpF (Pub)
Boundary to Reject H0	LD (OF)
Boundary to Reject H1	Gm (-2)
Difference of Means (δ_1)	0.9
Standard Deviation (σ)	2.57
Maximum Sample Size	270

Simulation Boundaries						Latest	Overall Simulation Results					
Look	Boundary					Simulated	Avg.	Avg.Sample	# Rejecting	# Rejecting	Total Simulations	
#	Samp Size	H0-	H0+	H1-	H1+	Test Stat.	Information	Size	H0	H1	Count	%
1	175.26		2.5469		0.9365	2.2230	6.76	175.00	4095	867	4962	49.62%
2	269.64		1.9896		1.9896	2.5439	10.25	270.00	3910	1128	5038	50.38%
3												
4												
5												
6												
7												
8												
9												
10												
11												
12												
						**Total**	8.52	222.86	8005	1995	10000	
						**%**			80.05%	19.95%		

Design Outputs	
Max. Sample Size (Nmax)	270
Max. Information (Imax)	10.2060

Simulation Parameters	
Simulation Scale	Sample Size
Difference of Means	0.9
Standard Deviation	2.57
Number of Trials	10000
Refresh Every 'n' Trials, n =	1000
Simulation Starting Seed	Clock

Run | Single Step | Reset | Stop

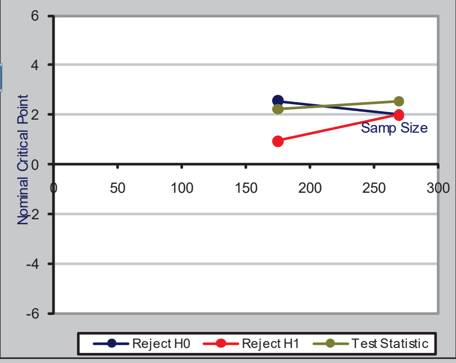

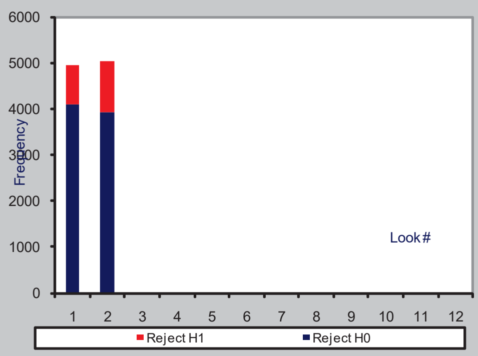

Trial # = 10000
Simulation Seed = 38273
Elapsed Time = 0:00:12

5.9 Adaptive Design and Sample Size Re-estimate

The current sample size estimate is based on numerous assumptions. The main assumption affecting the sample size is that of the true effect size and the standard deviation for the difference in the mJOA. These assumptions will be verified during the study and sample size readjustment will be performed if needed, using the adaptive techniques. The sample size readjustment will be performed after the first interim analysis of the data, currently planned at 65% data available at 180 days. The details of the adaptive change will be described in a separate Statistical Analysis Plan (SAP).

5.10 Analysis Populations

For definitions of subjects' status in the study see Section 4.6 Subjects Completion and Disposition.

5.10.1 Intention-to-Treat

Consenting patients who qualify and are randomized to a study arm (i.e. enrolled subjects) will be included in the Intent-To-Treat (ITT) analytic sample primary analysis population, regardless of the actual treatment received. Screen failures will be recorded on a screening eCRF and will not be included in the ITT group. Values for the patients who do not have the 6-month end point will be imputed to create complete ITT population.

5.10.2 Modified Intention-to-Treat

The modified Intent-To-Treat (mITT) population is defined as all consenting patients who receive study-directed treatment and have any follow-up. The mITT population will be used for safety analyses.

5.10.3 Per-protocol Population

Enrolled subjects who receive at least 80% of the treatment medication (riluzole or placebo) and receive the surgery will form per-protocol population.

5.10.4 Completed Subjects population

Subjects in the per-protocol population who complete 180 days follow-up will form Completed Subjects Population.

5.10.5 Primary analysis population

Primary efficacy analysis will be performed on ITT population. Primary safety analysis will be performed on mITT population.

5.11 Randomization

The randomization will be performed at 1:1 ratio between the investigational and the control arm. Sequenced and sealed envelopes or a similar randomization approach will be given to participating centers. Randomization will be carried out according to the blocks procedure. Block randomization is used to assign eligible patients to the treatment arms in order to avoid serious imbalance in the number of participants assigned to each group within a center. The pattern of the blocks will be concealed to avoid selection bias. Two block sizes will be used, with 2 and 4 subjects per block. Block sizes will be chosen at random. The order of block sizes will be randomly shuffled. This scheme makes breaking the blinding by working out the block pattern extremely difficult and reasonably protects against biased allocation of patients.

6 Risk Analysis

6.1 Risks

The risks that occur in this study are associated with the general risks of cervical spine surgery and study-specific risks associated with the use of investigational treatment.

Anticipated AEs which may occur as a direct result of the treatment or general anesthesia are identified and listed below. These risks will be present regardless of the participation in the trial.

1. Death
2. Pseudoarthrosis
3. Hardware failure
4. Screw malposition
5. Nonunion
6. C5 radiculopathy
7. Axial pain (nuchal or periscapular pain or neck fatigue)
8. New neck pain
9. Adjacent segment degeneration, defined as the development of a new radiculopathy or myelopathy referable to a segment adjacent to a previously fused level
10. Spinal instability
11. Dural tear
12. Epidural hematoma
13. Deep infection
14. Superficial infection
15. Dysphagia
16. Dysphonia
17. New radiculopathy other than C5
18. Cardiopulmonary event
19. Stroke
20. Cortical blindness
21. Incision complications
22. Reactions to anesthesia

The most commonly observed AEs associated with the use of riluzole more frequently than placebo treated patients are:

1. Asthenia
2. Nausea
3. Dizziness
4. Decreased lung function
5. Diarrhea
6. Abdominal pain
7. Pneumonia
8. Vomiting
9. Vertigo

10. Circumoral paresthesia
11. Anorexia
12. Somnolence

Other side effects associated with use of riluzole may occur.

6.2 Actions to Minimize Increased Risks

The surgical procedures used in this study are considered standard of care. The investigational treatment of this study is an off-label use of approved drug. Study-related procedures involve non-invasive clinical examinations and patient questionnaires.

Risks to the patients are managed by:

- Use of an FDA and Health Canada approved drug with known general side effects profile
- Interim efficacy analysis to determine early stop in the case of futility
- Centralized medical monitoring of safety events
- Procedure for braking the blind assignment in the case of safety concern

7 AE Reporting

AE information will be collected throughout the study and will capture all AEs, anticipated or unanticipated. The Investigator or Research Coordinator will record all AEs on the appropriate CRF. The applicable CRF will capture the date of onset, severity, duration, outcome and relationship to the Investigational Drug or protocol.

Any event considered serious or unanticipated in nature, degree or severity must be reported to the Sponsor's representative within 24 hours of becoming aware of the adverse event.

All AEs must be followed until resolution or a stable clinical endpoint is reached. All required treatments and outcomes of the AE must be recorded.

All AEs and SAEs must be followed until:

- AE is resolved and has returned to normal/baseline values or has stabilized
- Subject is lost to follow-up or has withdrawn consent
- AE is judged by the investigator to be no longer clinically significant
- Subject has completed study required follow-ups (12 month visit)
- Study Closure

7.1 AE Definitions

7.1.1 Adverse Event

An AE is any unfavorable and unintended sign (including an abnormal laboratory finding), symptom, or disease temporally associated with the use of an investigational product, whether or not it is related to the investigational product and/or any unfavorable clinical event which impacts, or has the potential to impact, the health and/or safety of a clinical study participant. Events may be: complications; observations; change in subject condition; or, (un)related death. An AE may occur at any time the subject is participating in the study and is not limited to the period of exposure to a study procedure or exposure to the study drug.

7.1.2 Anticipated (Expected) Adverse Events

These are risks or events reported in the Investigator's Brochure and listed in the consent form. The AEs will be considered as "anticipated" or "expected" only if it is discussed in the protocol and included in the Informed Consent document.

7.1.3 Unanticipated (Unexpected) Adverse Events:

An unanticipated adverse event is any unexpected untoward event or medical occurrence in a study subject that is not consistent with the known, predicted possible effects of the research protocol. An unanticipated adverse event can therefore be any unanticipated, unfavorable, and unintended sign (including an abnormal laboratory finding), symptom, or disease temporally associated with the study that was not listed in the protocol, consent form or investigator's brochure. This includes any experience that suggests a significant hazard, contraindication, side effect, or precaution. In addition to this definition, any adverse event not included in the Informed Consent document should be considered

as a risk to be "unanticipated" or "unexpected." [Adapted from HHS & FDA 21 CFR 312.62 (6), 21 CFR 50.27 (a), 21 CFR 312.32 (a), FDA Docket No. 93N-0181].

7.1.4 Serious Adverse Event

A serious adverse event (SAE) is an AE that leads to death or serious deterioration in the health of the subject. SAE include events that:

- Lead to a death
- Lead to a serious deterioration in the health of the subject that
  - Results in a life-threatening illness or injury
  - Results in a permanent impairment of a body structure or a body function
  - Requires subject hospitalization or prolongation of existing hospitalization
  - Results in medical or surgical intervention to prevent permanent impairment to body structure or a body function
  - Results in fetal distress, fetal death or a congenital or abnormality/birth defect

The investigator will report any and all SAEs to the IRB\REB as required by the IRB\REB.

7.2 Study Termination or Suspension

If the Study Investigative Team determines that the study should be suspended or terminated for any reason, subjects will be promptly informed. A letter with a detailed explanation of the termination or suspension will be provided to the IRBs.

8 Investigator Responsibilities

8.1 Investigator Qualifications

Investigator shall have proper medical qualification and training to perform clinical duties involved in the trial. Further, investigator shall have Human Subjects Research Protection training. Finally, Investigator shall allocate sufficient time to perform duties involved in this clinical trial, to delegate duties to qualified research staff, to supervise the research team. And, to maintain the facility qualified for the study.

8.2 IRB\REB Approval

This study must have initial and continuing approval (at least annual) from an Institutional Review Board (IRB) or Research Ethics Board (REB) responsible for approving clinical studies. This can be a local or central IRB\REB.

Furthermore, screening or enrollment of subjects into the trial will not commence until the IRB\REB approval letter is received by the Sponsor. In addition, a copy of the IRB\REB approval letter must be filed on-site in the Investigator's study binder. Where appropriate, amendments to the protocol will be submitted for IRB\REB review and approval before implementation.

8.3 Protocol Adherence

The Investigator(s) agree to conduct the study in accordance with this protocol. Prior to beginning of the study, the Investigator(s) must sign the Investigator Agreement and the Protocol Signature Page of this protocol.

An Investigator must not make any changes in the study without first receiving approval in writing from the Sponsor and IRB\REB, except when necessary to eliminate apparent immediate hazards to a subject.

8.3.1 Review of Source Documents

The Investigator(s) agrees that the Sponsor's employees or designees, as well as FDA and Health Canada designees will have the right to audit and review pertinent medical records relating to this clinical trial.

8.3.2 Record of Investigational Drug Inventory

The Investigator(s) will maintain a Drug Accountability Log of all investigational products received, used, or returned during this study. The Drug Accountability Log should be available during monitoring visits. All investigational products not used in this study must be returned to the Sponsor before or at the completion of the study or at the Sponsors request.

8.3.3 Data Recording and Record Retention

1) All data will be recorded on electronic Case Report Forms (eCRF) for each subject enrolled in the study.
2) The Sponsor or Sponsor's designee will review completed eCRF, along with source documentation. The Investigator(s) will ensure that the medical records are made available for review by the study monitor, FDA or Health Canada, as required.

3) All subject study records are to be maintained in a secure storage facility for at least two years or for a longer period as required by the local regulations. Subject study records will not be destroyed before notifying the Sponsor that the records may be discarded. This includes the following documentation:
   a) Source Worksheets, Informed Consents, and other study logs and forms
   b) Investigational Product Accountability Logs and Investigational Product shipment receipts of all products shipped to the site
   c) Correspondence with the IRB\REB, Sponsor, FDA/Health Canada, Monitor, or other Investigators
   d) Study protocol and any amendments issued
   e) Protocol and Informed Consent approvals from the IRB\REB
   f) Clinical Study Agreement and curricula vitae of Investigator(s), and the Study Delegation Form.

8.3.4 Notification Reporting

The Investigator(s) is responsible for all reporting required per the IRB\REB.

9 Study Data Reporting and Processing

9.1 Study Data Collection

The electronic Case Report Form (eCRF) is designed to accommodate the specific features of the trial design. The eCRF contains data items specified in this Investigational Plan

All forms and other study related materials should be submitted according to the schedule reflected in Table 6.

Table 6: Schedule for Case Report Form and Reports Submission

Event	CRF's & Reports	Submission Schedule
**Screening**	Screening Assessment Forms	Within 1 week
**Enrollment**	Enrollment form	Within 1 week
**All eCRF forms**	Entered into EDC System within 14 calendar days	Within 2 weeks
**Other Forms**	Narrative Summaries, Protocol Deviation, Protocol Violation, Study Drop-out etc.	Within 2 week
**UAE/SAE/Un-blinding Reports**	UAE / SAE Report /Un-blinding Report Form	Within 24 hours

9.2 Site Data Monitoring and Quality Control

Primary data collection based on source-documented hospital chart reviews, source worksheets and subject interviews will be performed by investigator or study coordinators at each clinical site. Source data will be transferred onto Electronic Data Capture System (EDC) in an expedited fashion and latest within 14 calendar days of the form collection.

Data entered into the EDC will be continuously verified by the Sponsor/designee staff. Deficiencies identified will be communicated regularly by electronic requests for clarifications and corrections. In addition, frequent communication between the Study Coordinator(s) and the CRO will take place.

All clinical sites will be monitored periodically by the Sponsor/designee personnel for protocol adherence, adherence to CRO's Standard Operating Procedures (SOPs), accuracy of source worksheet completion, and compliance to applicable regulations. Corrective actions will be requested in the case of non-compliance.

In the initial phase of the trial, the Sponsor/designee will coordinate and host teleconference calls between the monitor, data management and each clinical site, as necessary, to resolve any problems concerning the protocol and data collection. Every effort will be made to ensure compliance with the protocol. In addition, Sponsor representative(s) will maintain personal contact with the investigator and staff throughout the study by phone, mail, email and on-site visits.

A weekly recruitment status report generated by the master tracking system will identify variations in recruitment frequency among sites. The overall recruitment will be evaluated against the targeted recruitment dynamic.

9.2.1 Subject Coding

Documents sent to the Sponsor/designee will not contain subject names. Each subject will be assigned a unique subject code—SIC. The Subject Code will consist of 8 characters in an alphanumeric combination. The site will maintain the link between the Subject Code and the names. An example is shown below in Table 2.

Table 7: Example of the Subject Code

Site Code			Connector	Subject Study Number			
A	B	C	-	n	n	n	n

Personal information may be reviewed for the purpose of verifying data in the SWs. This can be performed by the monitor, authorized Sponsor representative, regulatory agencies or quality assurance personnel. Personal medical information will be treated as confidential at all times.

9.3 Data Processing and Quality Control

The study will use a specialized electronic CRF data collection system (EDC) called OpenClinica®. OpenClinica® is a 21 CFR Part 11 compliant software solution to store the clinical data. Conventional data verification sub-routines will be programmed to test entry and logical errors, while all individual (subject-based) CRFs will be linked for cross-reference. Periodic analysis of each data field across cases will be performed in order to examine the expected distributions and to identify outliers for possible mistakes.

9.3.1 Data Cleaning

All eCRFs will be subjected to initial inspection for omitted data, gross data inconsistencies, and timeliness of reporting. Any deficiencies will be resolved using electronic tracking and revision of errant forms at the clinical site.

9.3.2 Data Entry

Data entry will be directly into the EDC system from source worksheet and will be performed at the investigative sites. All eCRF data entry screens are similar to the corresponding study worksheets in order to reduce transcription error by data entry personnel.

9.3.3 Data Editing

Each data record is evaluated with an extensive electronic intra-form and inter-form edit checking on a regular interval. Any discovered error is then referred to the clinical site for review and correction by either the Study Coordinator or Investigator. Only the Investigator and Study Coordinator are authorized and allowed to edit and modify the data. Sponsor/designee staff does not have access rights to change the stored study data. The clinical database system stores history of all changes made to the data, with electronic time and date stamp and electronic signature. Once entered, the data cannot be erased; it can be only edited.

9.3.4 Data Update

The cycle of data edit will be ongoing until all the data are clean. The Sponsor or designee will monitor the clinical site for source documentation verification. If further data entry or source documentation errors are discovered during the site visit the corrections will be made at that time.

9.3.5 Final Data Quality Analyses

All exported datasets for analyses will undergo a final data cleaning procedure using programmed logical routines unique to each exported dataset.

9.3.6 Data Form Inventory

A data form inventory system will be utilized to assure accurate record keeping and constant tracking of these items. Sites will be notified as necessary of all forms that are delinquent.

9.3.7 Data Back-up

The EDC system maintains continuous advanced mirroring back-up system on the secure operating servers. In addition, off-site full back-ups will be performed daily.

9.3.8 Report Generation and Summary Statistics

A customized report is generated for record keeping and subject scheduling, serving as an overview of the current recruitment, follow-up and data processing status.

9.4 Confidentiality and Protection of Study Files

Access to the EDC is protected with an industry strength passcode for each individual user. Passcodes are user-specific and protect confidentiality and the data by allowing variable levels of access. The data transmittal to the EDC database uses approved high industry standards for secure data encryption and transmission. Hard copies of source worksheets are kept in a locked, secure location when not in use.

10 Study Management

10.1 Operations Committee

Sponsor will appoint Operations Committee. The Operations Committee will approve the final trial design and protocol issued to clinical sites. This committee will be responsible for general administrative management of the trial. This committee will meet as needed by conference or teleconference to monitor subject recruitment, clinical site progress, and protocol compliance. It will also be responsible for reviewing the final results, determining the methods of presentation and publication, and selection of secondary projects and publications.

10.2 AOSpine North America Methods Core

AOSpine North America Methods Core unit will be responsible for supervising trial operations, in particular clinical data management and operations of EDC system.

10.3 Monitoring

10.3.1 Pre-Investigation Visits (Site Evaluation Visit)

The Sponsor will ensure that the Investigator clearly understands and accepts the obligations incurred in undertaking a clinical investigation. Prior to the initiation of a clinical investigation, the Monitor or Sponsor representative may visit the clinical site to ensure that the Investigator:

- Understands the nature of the protocol or investigational plan
- Understands the requirements for an adequate and well-controlled study
- Understands and accepts the obligation to conduct the clinical investigation in accordance with applicable sections of Title 21 of CFR or any other applicable regulation
- Understands and accepts the obligation to obtain informed consent in accordance with 21CFR Part 50
- Understands and accepts the obligation to obtain IRB\REB review and approval of a clinical investigation before the investigation may be initiated, and to further ensure a continuing review of the study by the IRB\REB in accordance with 21CFR Part 56, and to keep the Sponsor/designees informed of such IRB\REB approval and subsequent IRB\REB actions concerning the study
- Has access to an adequate number of suitable subjects to conduct the investigation
- Has adequate facilities and staff for conducting the clinical investigation
- Has sufficient time from other obligations to carry out the responsibilities to which the investigator is committed by applicable regulations

10.3.2 Periodic Visits

The Sponsor will assure throughout the clinical investigation that the Investigators' obligations, as set forth in applicable regulations and in GCP guidelines, are being fulfilled and that the facilities used in the clinical investigation continue to be acceptable. Personal contact between the Monitor and the Investigator will be maintained throughout the clinical investigation. The monitor will visit the Investigator at the site of the investigation frequently enough to ensure that:

- Facilities/staff used by the Investigator continue to be adequate for purposes of the study
- The study protocol or investigational plan is being followed
- Changes to the protocol have been approved by and reported to the IRB\REB and/Sponsor
- Accurate, complete, and current records are maintained
- Accurate, complete, and timely reports are made to Sponsor and the IRB\REB
- The Investigator is carrying out the agreed upon activities and has not delegated them to other unspecified staff
- The accuracy of the data, individual subject records and other supporting documents will be compared to the reports prepared by the investigator for submission to the Sponsor. During a periodic visit, therefore, the Monitor will compare a representative number of subject records and other supporting documents with the Investigator's reports to determine that:
    - The information recorded in the Investigator's reports is complete, accurate, and legible
    - There are no omissions in the reports of specific data elements
    - Missing visits or examinations are noted in the reports
    - Subjects failing to complete the study and the reason for each failure are noted in the reports
    - Informed consent has been documented in accordance with Parts 50 and 56

10.3.3 Record of On-Site Visits

The Monitor will maintain a record of the findings, conclusions, and action taken to correct deficiencies for each on-site visit. The record will include the date of the visit, the name and address of the Investigator, any findings, conclusions and any actions taken to correct deficiencies.

10.4 Direct Access to Source Documentation

Direct access to source documentation must be allowed for the purpose of verifying that the data in the EDC are consistent with the original source data. Findings from this review of source worksheets and source documents will be discussed with investigational staff. The Sponsor expects that the relevant investigational staff will be available, the source documents will be accessible and a suitable environment will be provided for review of study-related documents during monitoring visits. The monitor will meet with the Investigator on a regulator basis to provide feedback on the conduct of the study.

11 Document Control

11.1 Protocol

11.2 Protocol Amendments

No changes to the IRB\REB-approved protocol are allowed except when removing immediate threats for patient safety, or when the change is purely of an administrative or logistical nature. Any change to the protocol made to protect the life and well-being of the enrolled subjects must be reported to the Sponsor or its representative within 5 days.

Protocol amendments will be signed off on by the Sponsor.

11.3 Protocol Deviations

Protocol deviation is non-adherence to the protocol that does not involve inclusion/exclusion criteria, primary efficacy variable and GCP guidelines. Protocol deviations are minor and do not impact the study in a major way. Protocol deviations are to be reported to the Sponsor/CRO within 10 working days of being identified.

11.4 Protocol Violations

A protocol violation is any significant divergence from the protocol on the part of the patient, Investigator or Sponsor that affects inclusion/exclusion criteria, primary efficacy variable or GCP guidelines. The Medical Monitor and the Sponsor will be notified immediately. Violations will be recorded at the site and reported to the IRB\REB as required. The Sponsor/CRO shall be notified within 5 working days of occurrence.

11.5 Record Retention

In compliance with ICH/GCP guidelines, the investigator/institution will maintain all CRFs and source documents that support the data collected from each subject, as well as all study documents in ICH/GCP Section 8: Essential Documents for the Conduct of a Clinical Trial, and all study documentation as specified by the applicable regulatory requirement(s).

Clinical research records shall be stored in a manner that ensures privacy, confidentiality, security and accessibility of the records both during and after the conduct of clinical research/trial. The investigator/institution will take measures to prevent accidental or premature destruction of those documents. Essential documents must be retained for at least 2 years after study completion.

If the responsible investigator retires, relocates, or, for other reasons, withdraws from responsibility of keeping the study records, custody must be transferred to a person who will accept the responsibility. The Sponsor/CRO must be notified in writing of the name and address of the new custodian. Under no circumstance shall the investigator relocate or dispose of any study documents before having obtained written approval from the Sponsor.

If it becomes necessary for the Sponsor or appropriate regulatory authority to review any documentation relating to the study, the investigator must permit access to such reports.

12 References

Reference List

1. Ates O, Cayli SR, Gurses I, Karabulut AB, Yucel N, Kocak A, Cakir CO, Yologlu S. Do sodium channel blockers have neuroprotective effect after onset of ischemic insult? Neurol Res. 2007 Apr;29(3):317-23.

2. Ates O, Cayli SR, Gurses I, Turkoz Y, Tarim O, Cakir CO, Kocak A. Comparative neuroprotective effect of sodium channel blockers after experimental spinal cord injury. J Clin Neurosci. 2007 Jul;14(7):658-65.

3. Bensimon G, Doble A. The tolerability of riluzole in the treatment of patients with amyotrophic lateral sclerosis. Expert Opin Drug Saf. 2004 Nov;3(6):525-34.

4. Bensimon G, Lacomblez L, Meininger V. A controlled trial of riluzole in amyotrophic lateral sclerosis. ALS/Riluzole Study Group. N Engl J Med. 1994 Mar 3;330(9):585-91.

5. Heurteaux C, Laigle C, Blondeau N, Jarretou G, Lazdunski M. Alpha-linolenic acid and riluzole treatment confer cerebral protection and improve survival after focal brain ischemia. Neuroscience. 2006;137(1):241-51. Epub 2005 Nov 14.

6. Hugon J. ALS therapy: targets for the future. Neurology. 1996 Dec;47(6 Suppl 4):S251-4.

7. Hugon J. Riluzole and ALS therapy. Wien Med Wochenschr. 1996;146(9-10):185-7. Review.

8. Lacomblez L, Bensimon G, Leigh PN, Guillet P, Meininger V. Dose-ranging study of riluzole in amyotrophic lateral sclerosis. Amyotrophic Lateral Sclerosis/Riluzole Study Group II. Lancet. 1996 May 25;347(9013):1425-31.

9. Lacomblez L, Bensimon G, Leigh PN, Guillet P, Powe L, Durrleman S, Delumeau JC, Meininger V. A confirmatory dose-ranging study of riluzole in ALS. ALS/Riluzole Study Group-II. Neurology. 1996 Dec;47(6 Suppl 4):S242-50.

10. Landwehrmeyer GB, Dubois B, de Yébenes JG, Kremer B, Gaus W, Kraus PH, Przuntek H, Dib M, Doble A, Fischer W, Ludolph AC; European Huntington's Disease Initiative Study Group. Riluzole in Huntington's disease: a 3-year, randomized controlled study. Ann Neurol. 2007 Sep;62(3):262-72.

11. Lang-Lazdunski L, Heurteaux C, Lazdunski M. A word of caution in extrapolating the riluzole spinal cord injury protective effects obtained in a rabbit model under ketamine anesthesia. J Thorac Cardiovasc

Surg. 1999 Dec;118(6):1157.

12. Lang-Lazdunski L, Heurteaux C, Vaillant N, Widmann C, Lazdunski M. Riluzole prevents ischemic spinal cord injury caused by aortic crossclamping. J Thorac Cardiovasc Surg. 1999 May;117(5):881-9.

13. Miller RG, Mitchell JD, Lyon M, Moore DH. Riluzole for amyotrophic lateral sclerosis (ALS)/motor neuron disease (MND). Cochrane Database Syst Rev. 2007 Jan 24;(1):CD001447. Review.

14. Mu X, Azbill RD, Springer JE. Riluzole and methylprednisolone combined treatment improves functional recovery in traumatic spinal cord injury. J Neurotrauma. 2000 Sep;17(9):773-80.

15. Mu X, Azbill RD, Springer JE. Riluzole improves measures of oxidative stress following traumatic spinal cord injury. Brain Res. 2000 Jul 7;870(1-2):66-72.

16. Schwartz G, Fehlings MG. Evaluation of the neuroprotective effects of sodium channel blockers after spinal cord injury: improved behavioral and neuroanatomical recovery with riluzole. J Neurosurg. 2001 Apr;94(2 Suppl):245-56.

17. Wang SJ, Wang KY, Wang WC. Mechanisms underlying the riluzole inhibition of glutamate release from rat cerebral cortex nerve terminals (synaptosomes). Neuroscience. 2004;125(1):191-201.

13 Appendices

13.1 Appendix A: Riluzole Drug Insert

13.2 Appendix B: Scales and Questionnaires

Scales:

mJOA

Nurick

ASIA

Questionnaires:

NDI

SF-36v2™

EQ-5D

Bazaz
