# Supplementary material for: Riluzole for Degenerative Cervical Myelopathy: A Secondary Analysis of the CSM-PROTECT Trial
Source: JAMA Netw Open. 2024 Jun 21;7(6):e2415643. doi: 10.1001/jamanetworkopen.2024.15643 (PMC11193126; doi:10.1001/jamanetworkopen.2024.15643)
Supplement: Supplement 2. — eFigure. Q-Q Plots of 1-Year Change From Baseline to Examine Data Normality eTable. Application of the GST in the Estimation of GTE [file jamanetwopen-e2415643-s002.pdf]

Supplementary Online Content

Fehlings MG, Pedro KM, Alvi MA, et al. Riluzole for degenerative cervical myelopathy: a secondary analysis of the CSM-PROTECT trial. *JAMA Netw Open*. 2024;7(6):e2415643. doi:10.1001/jamanetworkopen.2024.15643

eFigure. Q-Q Plots of 1-Year Change From Baseline to Examine Data Normality

eTable. Application of the GST in the Estimation of GTE

This supplementary material has been provided by the authors to give readers additional information about their work.

© 2024 Fehlings MG et al. *JAMA Network Open*.

eFigure. Q-Q plots of 1-year change from baseline to examine data normality. P-values were obtained from the Shapiro test.

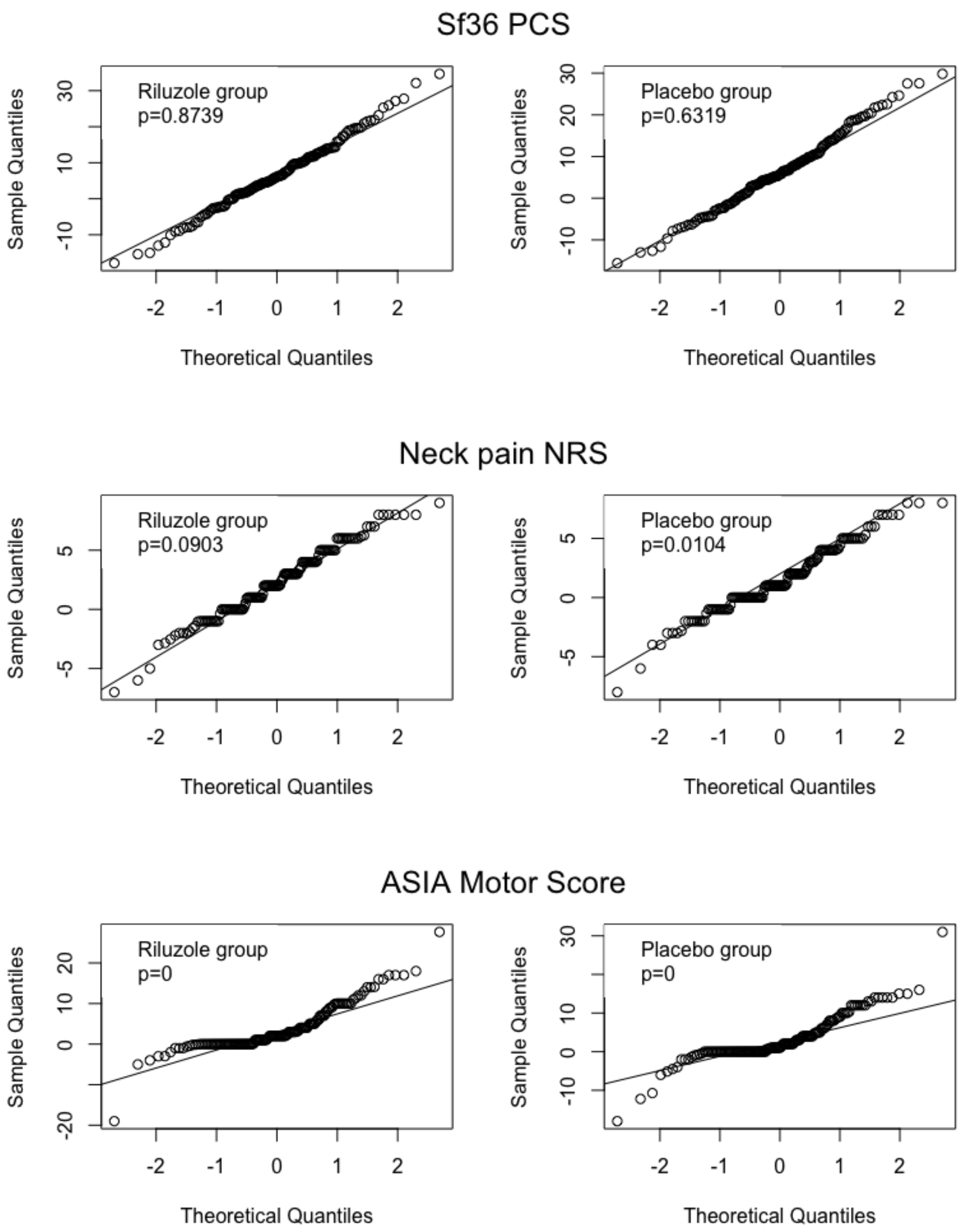

© 2024 Fehlings MG et al. *JAMA Network Open*.

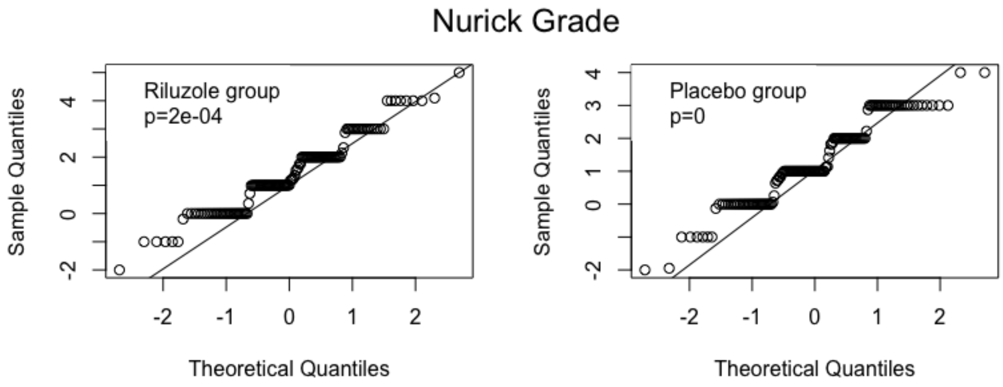

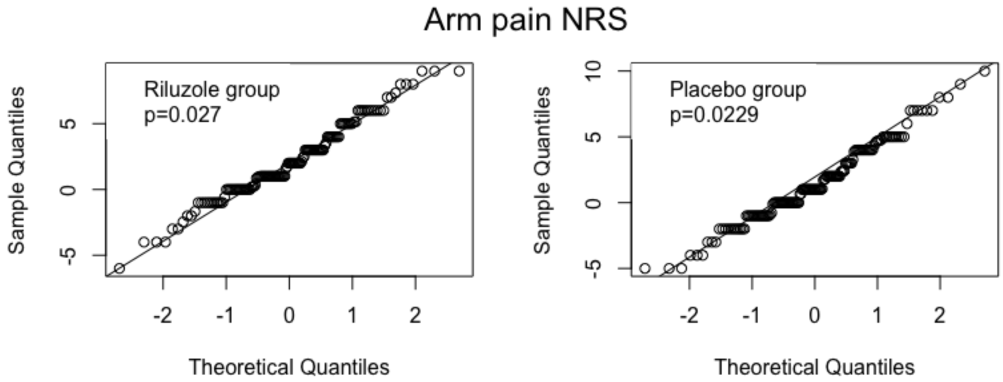

© 2024 Fehlings MG et al. *JAMA Network Open*.

eTable. Application of the GST in the Estimation of GTE

	Theta value			
	35 d	6 mo	1 y	
Arm Pain NRS	0.09	0.08	0.13	-
ASIA Motor Score	0.02	0.02	0.05	+
Neck Pain NRS	0.14	0.14	0.15	-
Nurick grade	0.01	0.04	0.05	-
SF-36 PCS	0.07	0.05	0.02	+
GTE (SD)	0.07 (0.04)	0.07 (0.04)	0.08 (0.04)	
Mean Rank sum (placebo)	704.10	703.20	698.80	
Mean Rank sum (riluzole)	752.30	753.20	757.80	
P value	0.04	0.04	0.02	

The GTE represents the mean of the five theta values at each time point. *P* values were derived using O' Brien's rank sum type test with variance adjustment for testing the general nonparametric Behrens-Fisher hypothesis. The SD represents a conservative estimate (upper bound) of GTE standard deviation. The sign indicates the direction of values prior to coding, with larger values signifying better outcomes in (+), while higher values indicate worse results in (-).

© 2024 Fehlings MG et al. *JAMA Network Open*.
