# Supplementary material for: Riluzole for Degenerative Cervical Myelopathy: A Secondary Analysis of the CSM-PROTECT Trial
Source: JAMA Netw Open. 2024 Jun 21;7(6):e2415643. doi: 10.1001/jamanetworkopen.2024.15643 (PMC11193126; doi:10.1001/jamanetworkopen.2024.15643)
Supplement: Supplement 3. — Data Sharing Statement [file jamanetwopen-e2415643-s003.pdf]

Data Sharing Statement

Fehlings. Riluzole for Degenerative Cervical Myelopathy. *JAMA Netw Open*. Published June 21, 2024. doi:10.1001/jamanetworkopen.2024.15643

Data
Data available: Yes
Data types: Deidentified participant data
How to access data: Data available upon reasonable request.
When available: With publication

Supporting Documents
Document types: None

Additional Information
Who can access the data: Researchers whose proposed use of the data has been approved
Types of analyses: For any purpose
Mechanisms of data availability: With a signed data access agreement approved by the investigators.
